# Understanding the feasibility of chemotherapeutic and immunotherapeutic targets against non-small cell lung cancers: an update of resistant responses and recent combinatorial therapies

**DOI:** 10.37349/etat.2023.00171

**Published:** 2023-10-10

**Authors:** Parth Malik, Ruma Rani, Raghu Solanki, Vishal Haribhai Patel, Tapan Kumar Mukherjee

**Affiliations:** Istituto Nazionale Tumori-IRCCS-Fondazione G. Pascale, Italy; ^1^School of Chemical Sciences, Central University of Gujarat, Gandhinagar 382030, Gujarat, India; ^2^Indian Council of Agricultural Research (ICAR)-National Research Centre on Equines, Hisar 125001, Haryana, India; ^3^School of Life Sciences, Central University of Gujarat, Gandhinagar 382030, Gujarat, India; ^4^Institute of Biotechnology, Amity University, Noida 201301, Uttar Pradesh, India; ^5^Institute of Biotechnology, Amity University, Kolkata 700156, West Bengal, India

**Keywords:** Lung cancer, immunotherapy, chemotherapy, non-small cell lung cancer, immune checkpoint inhibitors, adenocarcinoma, squamous cell carcinoma

## Abstract

Despite consistent progress in prompt diagnosis and curative therapies in the last decade, lung cancer (LC) continues to threaten mankind, accounting for nearly twice the casualties compared to prostate, breast, and other cancers. Statistics associate ~25% of 2021 cancer-related deaths with LC, more than 80% of which are explicitly caused by tobacco smoking. Prevailing as small and non-small cell pathologies, with respective occurring frequency of nearly 15% and 80–85%, non-small cell LCs (NSCLCs) are prominently distinguished into lung adenocarcinoma (LUAD) and lung squamous cell carcinoma (LUSC), subtypes. Since the first use of epidermal growth factor receptor (EGFR) inhibitor gefitinib for NSCLC treatment in 2002, immense progress has been made for targeted therapies with the next generation of drugs spanning across the chronological generations of small molecule inhibitors. The last two years have overseen the clinical approval of more than 10 therapeutic agents as first-line NSCLC medications. However, uncertain mutational aberrations as well as systemic resistant responses, and abysmal overall survival curtail the combating efficacies. Of late, immune checkpoint inhibitors (ICIs) against various molecules including programmed cell death-1 (PD-1) and its ligand (PD-L1) have been demonstrated as reliable LC treatment targets. Keeping these aspects in mind, this review article discusses the success of NSCLC chemo and immunotherapies with their characteristic effectiveness and future perspectives.

## Graphical abstract

**Graphical abstract.**
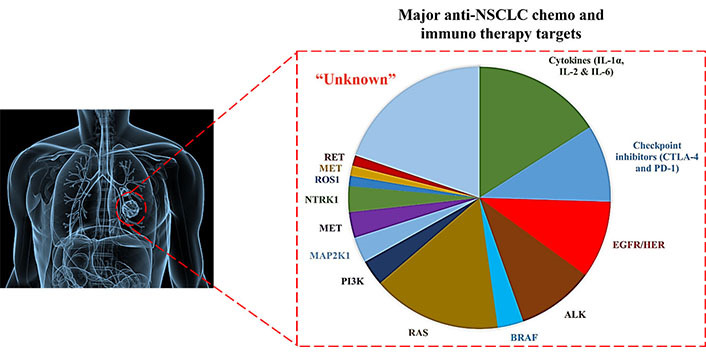
Quantitative diversity of gene mutations manifesting NSCLC pathogenic manifestation. RET: rearrangements during transfection; MET: mesenchymal-epithelial transition; ROS1: Proto-oncogene tyrosine-protein kinase; NTRK1: neurotrophic tropomyosin receptor kinase 1; PI3K: phosphatidylinositol 3-kinase; RAS: rat sarcoma; BRAF: v-RAF murine sarcoma viral oncogene homolog B1; ALK: anaplastic lymphoma kinase; HER: human EGFR; CTLA-4: cytotoxic T-lymphocyte-associated antigen 4; IL-1α: interleukin-1α

## Introduction

**Figure 1:**
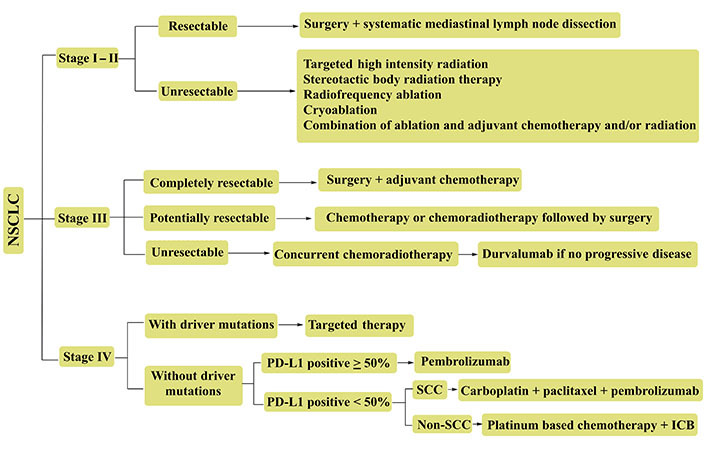
Representative NSCLC treatment recourses for various tumor stages. Advanced stage tumors (III & IV) are often treated with combinatorial therapies. PD-L1: programmed cell death-1 ligand; SCC: squamous cell carcinoma; ICB: immune-checkpoint blockade

Lung cancer (LC) prevails as a heterogeneous pathophysiology, contributing to ~25% of 2021 global cancer mortalities. Recent stats create a pondering herein, with 1.76 million deaths from the 2.09 million, 2018 sufferers [1]. The scenario is more vulnerable in the United States (US), with 1,35,720 deaths from the 2,28,820 fresh cases [2]. Histological distinctions of LC demarcate it as small cell LCs (SCLCs) and non-small cell LCs (NSCLCs) regimens. Pathologically, NSCLCs are more threatening, accounting for ~80–85% sufferers with lung adenocarcinoma (LUAD) and lung squamous cell carcinoma (LUSC) as prominent distinctions [3]. The stage specific NSCLC treatments, wherein advanced tumors are frequently dealt with prompt screening of underlying gene mutations, decide the singular or combinatorial chemo and immunotherapies (Figure 1).

The genetic basis of NSCLC is described by the mutations leading to inactivated tumor suppressor genes or conversion of proto-oncogenes to oncogenes. Most frequently mutated genes in NSCLC include epidermal growth factor receptors (EGFRs, 10–35%), Kirsten rat sarcoma (KRAS, 25%) and fibroblast growth factor receptor 1 (FGFR1, 9–20%) while anaplastic lymphoma kinase (ALK), mesenchymal-epithelial transition (MET) factor are the next significant. The others, phosphatidylinositol-4,5-bisphosphate 3-kinase catalytic subunit alpha (PI3KCA), v-RAF murine sarcoma viral oncogene homolog B1 (BRAF), Proto-oncogene tyrosine-protein kinase (ROS1), rearrangements during transfection (RET) collectively account for 1–4% NSCLC manifestations [4, 5]. Frequent *EGFR* involvement in NSCLC include a point mutation in exon 21 (~40% sufferers) besides exon 19 deletion (E746–A750), resulting in codon 858 substitution (L858R). Of note, oncogenic stimulators, *ROS1* and *ALK* rearrangements occur more frequently in never-smokers. The dearth of genetic mutations guards the tumor cells from the actions of cytotoxic T cells (T_c_) while other immunological precursors on tumor cells are impaired by the T regulatory (T_reg_) cells, causing a therapeutic failure [6, 7]. Thereby, understanding the gene mutations *vis-à-vis* gender and smoking variability could significantly improve the success of targeted therapies via timid detection and enhanced accuracy.

Current NSCLC therapies are distinguished as generalized chemotherapy, targeted therapy and immunotherapy, wherein chemotherapy kills the cancer cells by inhibiting their proliferation and growth. Effective at all stages, chemotherapy proceeds for a fixed number of cycles over a designated timeline. The Food and Drug Administration (FDA)-approved chemotherapeutic drugs (CDs) for NSCLC are carboplatin, cisplatin, docetaxel, etoposide, gemcitabine, paclitaxel, vinorelbine and pemetrexed. Persistent concerns with chemotherapy comprise its dosage vulnerability and damage to healthy cells, majorly from blood, skin, and neurons. These challenges are amicably addressed in targeted therapy whereby specific genes, proteins, or signaling pathways, aggravating the tumor growth and survival, are aimed. Though targeted therapy is optimized *vis-à-vis* singular or multiple targets, most of the CDs exhibit resistance within a few months of treatment. The significance of this claim is well-demonstrated by the varied tyrosine kinase inhibitor (TKI) generations, wherein first-generation drugs exhibited success for exon 19 and exon 21 point and frame-shift EGFR mutations [8]. Of note, EGFR is a protein tyrosine kinase. Subsequent resistant manifestations arising from genetic and signaling irregularities unravelled the T790M (substituting threonine with methionine) mutation, hampering the target binding efficacy of the inhibitor. Multiple 2nd and 3rd generation TKIs (gilotrif, dacomitinib, HM61713, CO-186) counter this resistance with the tedious dosage monitoring being resolved in the T790M mutation-specific, 3rd generation drugs. The T790M exclusive success of 2nd generation TKIs has been the major reason for their selective administration. Treatment with 3rd generation TKIs has been reported for resistance via C797S (cytosine to serine) mutation-impaired drug binding. Recent studies demonstrate a resistive response towards EGFR-TKIs for the cis conformation towards the 1st and 3rd generation TKIs co-delivery [9–11]. Over the past few years, emergence of 4th generation TKIs has countered the resistance even further.

Opposed to chemotherapy, immunotherapy has witnessed significant success for NSCLC treatment, in particular for advanced stages. The immunotherapies are programmed to forbid the recognition of tumor cells as native, by pertinent T_reg_ cell actions and tumor-associated macrophages (TAMs), via optimum T_c_ actions, attenuated T_reg_ and TAM actions or immune-checkpoint inhibitors (ICIs). Additionally, treatment via immunotherapy comprises modulated cytokine functioning such as interleukins (ILs, e.g., IL-1α and IL-2) or cytotoxic T-lymphocyte associated antigen-4 (CTLA-4), and programmed cell death 1 (PD-1) actions [12]. A cautious look at the literature reveals multiple clinical trials being initiated using ILs and their combinations with specific gene targeting CDs and PD-1/PD-L1 inhibitors but till date none of these has emerged statistically significant. Targeting PD-1/PD-L1 inhibitors have witnessed significant success in phase II and III clinical trials, with nivolumab, pembrolizumab (towards PD-1) and atezolizumab, drvalumab and avelumab (towards PD-L1) as medications [13, 14]. Like chemotherapy, immunotherapy also exhibits resistance against various NSCLCs and several novel approaches have been optimized to overcome resistance [15, 16]. Besides the chemo and immunotherapies, targeted gene therapy supersedes the mutational uncertainties, receiving recent interest *vis-à-vis* clustered regularly interspaced short palindromic repeats (CRISPR)/CRISPR-associated (Cas) and similar assays [17, 18]. Keeping the above aspects in mind, the present review article sheds light on the latest successful NSCLC therapies about modulated activities of specific oncoproteins and immunomodulatory molecules. The information described herein could enrich the database for further reliable NSCLC treatment development.

## Noted targets in chemotherapy treated NSCLCs

Rising NSCLC mortality is majorly attributed to the late detection, with more than 50% positive cases being already progressed to advanced stages when diagnosed. It is therefore highly urgent to improve the specificity of targeted therapies, necessitating a sound knowledge of perturbed gene expressions. This section, therefore, describes the CDs to the prominent NSCLCs aggravating protein expressions from *EGFR*, *KRAS*, *ALK*, *MET*, *ROS1*, *BRAF*, neurotrophic tropomyosin receptor kinase (*NTRK*), and *RET* genes. The mutations of these genes followed by enhanced expression or altered ligand binding are the prominent reasons for NSCLC complication. The following paragraphs, thereby, discuss the functional diversity and the confrontation of altered gene expression *vis-à-vis* FDA-approved CDs.

### The EGFR

The *EGFR* or *ErbB1* or human EGFR 1 (*HER1*) prevails as a member of receptor tyrosine kinases (RTKs) which are involved in the activation of mitogen-activated protein kinase (MAPK) and phosphatidylinositol 3-kinase (PI3K)-protein kinase B (AKT)-mammalian target of rapamycin (mTOR) signaling pathways, implicated in cell growth, proliferation and survival. Three major mechanisms in *EGFR* activation of these cell signaling pathways are aggravation of EGFR mutations in the malignant cells, enhanced expression of EGFRs, and finally either mutation of the specific target/ligand [epidermal growth factors (EGFs)] or their enhanced activities. The mutated receptor (EGFRs) and ligand (EGFs) fail to dissociate on releasing pro-survival and growth-promoting signals causing uncontrolled growth [19–21].

Statistically, the prevalence of *EGFR* oncogene mutations is 50% among Asian adenocarcinoma sufferers and 15% in the western patients. The two most common mutations of this gene are exon 19 deletion (60%) and L858R mis-sense mutations at position 858 (35%), wherein leucine is substituted by arginine, resulting in non-ligand bound *EGFR* activation. To date, erlotinib, gefitinib (1st generation), afatinib, dacomitinib (2nd generation), and osimertinib (3rd generation) are the FDA-approved drugs for *EGFR* inhibition (Table 1). Despite potent therapeutic programming, studies decipher a resistant manifestation for treatment with these TKIs, regulated via T790M secondary mutations, activation of erstwhile *EGFR* pathways and histological transformation [22, 23].

**Table 1:** Summary of NSCLC EGFR-TKI drugs with the targeted gene mutations and the working mechanisms

**Drug(s)**	**FDA approval specifications and targeted mutations**	**Generation**
Erlotinib, gefitinib, icotinib	Erlotinib: approved in May 2013 for 1st line metastatic and in November 2004 for 2nd line metastatic NSCLC treatment (efficient for exon 19 and L858R mutated NSCLC) Gefitinib: approved in July 2015 for 1st line metastatic NSCLC. Was earlier approved in 2003 for 3rd line but got retracted in 2005 (diarrhoea and liver dysfunction were noted as adverse effects) Icotinib was approved in China in June 2011 and caused fewer AEs than gefitinib (61% against 70%), phase III double-blind study revealed a 3–4 months PFS	First
Afatinib, dacominitib	Afatinib got approval in July 2013 for 1st line metastatic, T790M mutant patients & in January 2018 for 1st line metastatic non-resistant mutations Dacomitinib was approved in September 2018 for 1st line, metastatic NSCLC patients	Second
Osimertinib, rociletinib, olmutinib, lazertinib	Osimertinib was approved by FDA in November 2015 for treating T790M mutated NSCLC besides controlling CNS metastasis Rociletinib was screened as active in T790M mutated NSCLC in clinical trials but was not approved by FDA due to its limited efficacy Olmutinib got FDA approval in December 2015 for T790M mutation harboring NSCLC Lazertinib got its first FDA approval in January 2021 for L858R, exon 19 deleted and T790M mutated advanced, metastasized NSCLC	Third with CNS penetration
1296P BLU-945, MA07.09 BBT-176, BPI-361175	1296P BLU-945 is efficient in treatment of T790M and C797S mutated, advanced NSCLC MA07.09BBT-176 was manageably toxic for 20–600 mg intake on daily basis, emerging suitable to treat exon 19 deleted, T790M & C797S mutated, advanced NSCLC BPI-361175 prolonged the life span of exon 19 deleted & T790M/C797S mutated NSCLC in mice models with significant control of CNS metastasis. Currently, it is being evaluated in phase I clinical trials in China & US	Fourth

AEs: adverse events; PFS: progression-free survival; CNS: central nervous system

*EGFR* mutant NSCLC is characterized by a pathology wherein unlike the *de novo* T790M, the T790M prevails via exon 19 deletions than in L858R mutated EGFR-TKI resistant sufferers [24]. For selective TKI pressure, this mutated clone predominates. Perhaps, this eventuality led to the discovery of osimertinib, an EGFR-TKI that potently aggravates its sensitization as well as T790M resistant mutations. Studies have demonstrated improved outcomes for osimertinib therapy of T790M mutated NSCLCs besides enhanced PFS (18.9 months) and overall survival (OS, 38.6 months) in first-line settings [19, 20]. Recently, several studies reported osimertinib resistance, suggesting its resolving as a priority [21, 25, 26]. The *EGFR* mutated NSCLCs are normally treated with varying generation TKIs, depending on the specific mutation intensity. Several investigations have elucidated a greater efficacy of combinatorial treatment, comprising varied stoichiometries of EGFR-TKIs and CDs. A phase II study evaluated the osimertinib safety and efficacy via chemotherapy combination, for T790M mutation harboring NSCLC sufferers. Even though tolerable extents were improved, no progress in survival duration was noticed, making the investigators pursue a re-investigation [27]. Another phase III study evaluated osimertinib efficacy with and without platinum-pemetrexed chemotherapy, in *EGFR*-mutated advanced NSCLC sufferers. Except for minor AEs, the combination exhibited a manageable tolerance, making the analysis feasible for further studies [28].

#### Post-therapeutic resistant outcomes to EGFR targeted therapies

Despite a long history of EGFR-TKIs treated *EGFR*-mutant NSCLC, most outcomes have been transient, resulting in relapse post 6 months of initial treatment. The resistance could be *de novo* or in response to targeted agents’ exposure, prevailing as a resistant clone in single/multiple tumor cells. The manifestation of “acquired resistance” is quite frequent, either via secondary mutations or activation-independent pathways. Concurrent replacement of threonine (T790M), the well-demonstrated *EGFR* gene mutation, alters the tyrosine kinase domain in the native configuration, enhancing the affinity for ATP over the first-generation reversible TKIs [29]. The second frequent resistant outcome involves *MET* amplification (amp) driven by PI3K-AKT-mTOR signaling [30]. Other prominent outcomes comprise mutations in *PI3KCA*, *HER2*, *BRAF*, signal transducer and activator of transcription 3 (STAT3), AXL kinase, Crk-like protein (CRKL) amp and 5% unexpected transformation to SCLCs [31]. Resistance to targeted chemotherapy could be approached more authentically via identification of aberrant pathways. For instance, afatinib (a second-generation irreversible TKI), covalently binds to EGFR/HER1, HER2 and counters the T790M mutation with a 7% overall response rate (ORR) and enhanced PFS from 1.1 months to 3.3 months than placebo [32]. Presently, dual EGFR blockade with EGFR-TKIs and cetuximab is being examined [33]. Combined MET and T790M inhibition has been successful in murine models, currently screened in humans with a MET/ALK inhibitor (crizotinib) in combination with an HER inhibitor (dacomitinib) [34]. Similarly, 3rd generation TKIs (CO-1868, AP26113) targeting T790M have countered the resistance and moderate the residual toxicity [35, 36]. Thereby, addressing resistance to targeted therapies is indeed possible; a future challenge pertains to screening optimal combinations *vis-à-vis* toxicity and expenditure prospects.

#### HER2

HER2 is viciously implicated in the activation of signaling pathways regulating cell proliferation and survival, on dimerization with other EGFR family members. Perhaps, the EGFR-HER2 heterodimerization induces a strong EGFR tyrosine kinase activation than its homodimerization [37]. A member of tyrosine kinase receptor family (HER1, HER3 and HER4 being others), HER2 accounts for ~3% LUAD cases [38]. Trastuzumab deruxtecan is the major drug used for the treatment of HER2 mutated advanced NSCLC, prevailing as antibody-drug conjugate (ADC). The working composition of trastuzumab-ADC carries an antibody that targets HER2 protein and is linked to a CD. Treatment with trastuzumab (drug conjugate) is usually adopted subsequent to being treated with at least one other drug.

The trials investigating trastuzumab-ADC efficacy for HER2 mutated NSCLC have mostly used trastuzumab in combinatorial mode. For instance, a recent phase II basket trial screened the adotrastuzumab emtansine efficacy in HER2-mutant LUAD patients, wherein 8 of the examined 18 (44%) patients responded. The median PFS (mPFS) was 5 months [39]. In a global retrograde investigation, 27 stage IV or recurrent HER2 mutated NSCLC sufferers were treated with afatinib, developing an ORR of 13% (3 of the 23 evaluable patients). The median OS from the stage of metastatic status confirmation was 23 months [40]. A phase II study on an erstwhile pan-TKI, pyrotinib (administered orally) that inhibits HER1, HER2 and HER4, screened 60 advanced NSCLC sufferers having HER2 exon 20 mutations. The examined subjects exhibited tumor progression subsequent to platinum chemotherapy and were administered 400 mg pyrotinib once, on a daily basis. Analysis revealed a 31.7% ORR with median response of 7 months. Although mPFS lasted 6.8 months, ~26.7% sufferers exhibited grade 3 treatment emergent AEs (TEAEs) [41, 42].

### Targeting KRAS

Nearly 30% NSCLCs exhibit a mutated rat sarcoma (*RAS*) gene. *RAS* is an acronym of a gene having three isoforms, i.e., KRAS, neuroblastoma rasviral oncogene homolog (NRAS) and Harvey RAS viral oncogene homolog (HRAS). The product of this gene acts as a small guanosine triphosphate (GTP) binding protein by guanine exchange factors (GEFs), is involved in diverse functions including proliferation, survival and differentiation through their GTP hydrolases (GTPases) [43]. In the GTP-bound state, KRAS activates the downstream signaling pathways such as MAPKs/extracellular signal-regulated kinases (ERKs) and PI3K-AKT-mTOR pathway, aiding in cell survival and proliferation. Mutations of *KRAS* are more common in adenocarcinoma and can be identified using next-generation sequencing (NGS) [44]. Most *KRAS* mutations are manifested via codon 12 substitution (G12C), gathering attention for NSCLC treatment [45]. Contrary to a higher G12C prevalence in smokers, the *KRAS* G12D is more frequent in non-smokers [46]. Present KRAS mutations exhibiting NSCLC therapies rely on the non-oncogene driven NSCLC, comprising immunotherapy with or without platinum chemotherapy. Clinically approved drugs for *KRAS* G12C are sotorasib and adagrasib. In phase I/II trial of sotorasib (Codebreak 100), 59 *KRAS*, G12C mutated, metastatic NSCLC patients treated with a median of 3 previous therapies, were given sotorasib orally. Analysis revealed 32.2% outcome with a confirmed objective response (complete or partial) in 88.1% cases. The mPFS was 6.3 months with grade 3 or 4 treatment-related toxicity in 11.6% patients. Inspection revealed a significant response in pretreated advanced tumors exhibiting G12C mutations [47].

Presently, sotorasib is being tested via randomized phase III, Codebreak 200 trial comparative to docetaxel (NCT04303780), with PFS and OS as the primary and secondary endpoints [48]. Adagrasib is another KRAS G12C-specific TKI, for which a phase I/II (KRYSTAL-1) trial involving 79 pre-treated NSCLC patients, administered 600 mg adagrasib, twice daily has been completed [49]. Of the 51 sufferers listed for response, 45% ORR was noticed with grade 1–2 side effects. Several erstwhile KRAS G12C inhibitors are being evaluated in phase I/II clinical trials (JNJ-74699157 and Gadolinia-Doped Ceria-6036) [50, 51]. As less than 50% sufferers responded positively to sotorasib or adagrasib, it could be presumed that some patients exhibit intrinsic resistance to KRAS G12C inhibition. This assumption gathers support from the preclinical observations [52]. Another source of resistance to KRAS targeted NSCLC therapies could be their unequal distribution across the tumor sites in a single patient [53]. Studies also demonstrate the emergence of adaptive resistance due to KRAS TKIs selective pressure, exhibited via amplified upstream drivers, including RTKs/tyrosine kinaseproto-oncogene (Src), homology 2 domain-containing phosphatase 2 which manifests from KRAS inhibition. Experimental evidence for reduced ERK activity via KRAS G12C TKIs does establish a suppressed ERK-driven RTKs/Src homology phosphotyrosine phosphatase 2 (SHP2) inhibition, further regulating NRAS, HRAS, and KRAS G12C to activate the MAPK/ERK signaling [52, 54]. It could be a further certainty that tumor cells may no longer depend on RAS pathway for survival and proliferation [55]. Therefore, despite inadequate clinical data, there is a high probability of KRAS G12C inhibitors being ineffective in many sufferers. Intriguing curiosity prevails for screening KRAS G12C inhibitors in combination with ICIs [56, 57]. In several preclinical studies, these combinations are being pursued as KRAS G12C positive tumor cell lines exhibit an immunosuppressive environment attenuated by KRAS inhibition [58, 59]. Other combinations led to the preclinical screening of adagrasib with SHP2 inhibitor, TNO155 [60]. However, no conclusive results have yet been published. Targeting with others is being attempted, prominently, MRTX1133, a KRAS G12D inhibitor [61]. Another molecule being examined is BI 1701963, a KRAS activator inhibiting the KRAS pathway irrespective of underlying mutation [62]. Of the other major KRAS targeting mechanisms, a messenger RNA (mRNA) vaccine targeting *KRAS* mutant cells has already entered a phase I trial, emerging potent from the immune response in the preclinical studies [63, 64].

### Anaplastic large cell lymphoma kinase

Rearrangements in anaplastic large cell lymphoma kinase (*ALK*) gene are witnessed in 2–7% advanced NSCLC sufferers, with a higher frequency in young non-smokers. The echinoderm microtubule-associated protein-like 4 (*EML4*) and *ALK* genes reside within the short arm of chromosome 2; inversion of which provided the novel fusion oncogene EML4-ALK [65, 66]. A mainstay in the transmembrane receptor kinases, ALK can activate multiple signaling cascades including PI3K-AKT, signaling adaptor protein crk-Guanine nucleotide exchange factor (Crk1-C3G), MAPK 2/3 MAPK 5 (MEK5)-ERK5, Janus kinase (JAK)-STAT pathways [67]. Major ALK alterations in NSCLC are characterized by point mutations, deletions and rearrangements leading to reactivation. The ALK rearrangements are manifested via interstitial deletion and inversion in chromosome 2p, resulting in EML4-ALK fusion gene product [68, 69]. Murine tumors and human cell lines expressing EML4-ALK have been screened towards ALK inhibitors [70, 71]. A striking observation herein has been the mutually exclusive (without *EGFR* and *KRAS*) prevalence of mutated *ALK* gene, illustrating a single gene product instigated malignant status [72, 73]. The frequent *ALK* rearrangements in LUADs are characterized by solid tumors and recurrent signet-ring cells rich in intracellular mucin, in the Western population [74]. Crizotinib is the first validated CD for targeted therapy of ALK-positive advanced NSCLC, enabling a 10.9-month PFS contrary to 7 months chemotherapy [75]. However, abysmal CNS penetration is the reason for crizotinib resistance, resolved using the 2nd generation ALK-TKIs (Table 2). The first amongst these is certinib, granted FDA approval in 2014 for ALK-positive NSCLC patients exhibiting crizotinib intolerance. First-line therapy approval for crizotinib was given in 2017 based on ASCEND-1 and ASCEND-2 studies [76, 77], with the latter accomplishing a 38.6% ORR to crizotinib pretreated ALK + NSCLC, via 750 mg daily intake [77]. A phase I, three-arm ASCEND-8 study showed that 450 mg certinib with food had a similar efficacy with much less gastrointestinal toxicity than 750 mg fasted, leading to its 450 mg OD approval with food [78]. However, subsequent randomized phase III trials, compared certinib with standard chemotherapy in first (ASCEND-4) and second-line (ASCEND-5) settings revealed multiple dose modifications due to AEs [79, 80].

**Table 2:** Summary of varied generation ALK-TKI drugs recommended for NSCLC treatment

**Drug**	**Month & year of approval**	**Circumstantial efficacy screened**	**Generation stage**
Crizotinib	August 2011	ALK metastatic tumors	First
Certinib	April 2014	ALK metastatic tumors	Second
Alectinib	December 2015	Progressed ALK metastatic tumors, exhibiting resistance to crizotinib	Second
	November 2017	ALK metastasis	
Brigatinib	April 2017	Progressed ALK metastatic tumors, displaying resistance to crizotinib	Second
Ensartinib	Could not be authentically traced	Exhibited 52% ORR in a phase II trial. A phase III trial found ensartinib better than crizotinib in systemic and intracranial diseases. ALK resistance were majorly due to G1269A, G1202R & E1210K mutations	Second
Lorlatinib	November 2018	ALK metastatic tumors treated with alectinib or certinib or crizotinib & at least one more ALK inhibitor	Third
TPX-0131	Currently being investigated	A compact macrocyclic inhibitor that fits well entirely in the ATP binding pocket. Efficient against compound mutations G1202R + L1198F, G1202R + L1196M, L1196M + L1198F, and G1202R + C1156F	Fourth
NVL-655	Currently being investigated	A brain penetrant small molecule inhibitor with significant potential against solvent drug resistance mutations, viz. G1202R, G1202R + L1196M and G1202R + G12269A	Fourth

Alectinib was the next screened ALK-TKIs, wherein ALEX trial inferred its preferential first-line treatment, ascertained via response of 303 untreated ALK-positive advanced NSCLC sufferers, randomized to receive alectinib or crizotinib. A 12-month analysis revealed a 68.4% survival for alectinib contrary to the 48.7% for crizotinib [81]. The observations were supported by 34.8-month mPFS for alectinib contrary to 10.9 months for crizotinib [82]. The mPFS with baseline CNS metastasis was 27.7 months with alectinib contrary to the 7.4 months 12-month PFS contrary to 43% for crizotinib. These observations were complemented by 71% ORR (60% for crizotinib) besides 78% intracranial response [83]. A further ALTA-1L study also supported the brigatinib efficacy, irrespective of ALK fusion variant or a tumor suppressor gene (TP53) mutation, in the sufferers with baseline brain metastasis [84]. Clinical efficacy of brigatinib (NCT02706626), is being analyzed for resistive ALK-mutations via treatment with other 2nd generation drugs [85]. A phase III trial (NCT03596866), evaluating brigatinib and alectinib efficacy in first-line ALK mutated NSCLC patients is currently ongoing [86]. The latest ALK-TKI being evaluated is ensartinib, a 2nd generation aminopyridine-containing small molecule reported for its MET, BAL, Axl, Ephrin type-A-receptor 2 (EPHA2), LTK, ROS1 and Ste20-like kinase (SLK) inhibitions [87]. A phase I/II study, comprising 97 advanced ALK-positive, NSCLC patients given 225 mg ensartinib daily, revealed rashness, nausea, vomiting, pruritus and fatigue as frequent aftermaths. The response rate and mPFS were 60%, 80% and 9.2 months, 26.2 months for ALK-TKIs evaluable and naive patients. Besides, sufferers exhibiting brain metastasis showed a 64% ORR [88]. A phase III clinical trial comparing ensartinib and crizotinib efficacy in ALK-positive NSCLC patients, neared its completion in 2018 [89].

A still potent, third-generation ALK and ROS1 inhibiting drug is lorlatinib, a small and compact macrocyclic inhibitor, restraining G1202R mutation but no compound aberrations [90]. A recent phase II trial observed a 47% ORR and a 63% objective intracranial response on 198 ALK-positive, advanced NSCLC sufferers with hypercholesterolemia, hypertriglyceridemia and peripheral neuropathy as prominent AEs [91]. A recent phase III CROWN analysis, comparing the lorlatinib and crizotinib efficacy showed a distinct PFS with 0.28 as hazard ratio, fetching lorlatinib the first-line FDA approval [92]. Current knowledge of lorlatinib is marred by inadequate comparative studies with 2nd generation drugs. Sequential use of “single mutant” active ALK TKIs, aggravates the risk of double ALK resistance, for which 4th generation ALK TKIs (TPX-0131, NVL-655) are being evaluated. TPX-0131 is a compact macrocyclic inhibitor that fits well in the ATP binding pocket and reduces the vulnerability to multiple ALK TKI-resistant mutations (solvent front, hinge region, gatekeeper, and compound mutations) [93]. NVL-655 is a brain penetrative small molecule inhibitor, confronting the solvent-dependent drug resistance (G1202R, G1202R-L1196M and G1201R + G1269A) [94]. Entrectinib and repotrectinib (TPX-0005) are the further potent ALK TKIs, specifically targeting tropomyosin receptor kinase (TRK) A/B/C, ALK, ROS1, and ALK, ROS1, TRK A/B/C [95, 96]. Results of phase I/II basket and STARTRK-1 trials reported adequate tolerance with selective potency for entrectinib [97]. Repotrectinib is a macrocyclic TKI, smaller than lorlatinib with a potent CNS activity. Its latest screening via TREDENT-1 study (NCT03093116) reveals an encouraging response in ALK mutant NSCLC [98].

#### Post-therapeutic mutations in ALK-positive NSCLC treatments

Like EGFR-TKIs, resistance has been reported for crizotinib-treated ALK-TKIs rearrangements. The inadequacy of crizotinib is majorly due to its abysmal CNS penetration [99]. The sufferers administered crizotinib, inevitably develop resistance, wherein L1196M, G11269A and C1156Y are the noted mutations that alter the ATP binding pocket structure and hinder the crozotinib binding with ALK [100]. Second-generation ALK-TKIs have been examined in distinct settings, emerging significant in treating crozotinib resistant ALK^+^, NSCLC sufferers. Lately, some concerns for alectinib have been reported via G1202R and I1171N/S/T mutations on-target resistance [101]. Though 3rd generation ALK-TKIs were proposed to minimize the resistance, a 2021 study demonstrated a prevalence of higher AEs for lorlatinib, in particular for aggressive CNS penetration [102]. TQ-B3139, WX-0593, PLB-1003, SAF-189, and CT-707 represent some novel ALK TKIs [103–107], whereas gilteritinib and XMU-MP-5 are currently under preclinical investigation. Several proteolysis-targeting chimeric degraders (PROTACs) are also being examined, programmed via advancing mechanism of 2nd generation drugs [108–110]. Concerns associated herewith include, kinase mutations and off-target effects [111]. Readers are suggested to refer 2022 review article by Peng and colleagues [87] to know more about some novel ALK-TKIs undergoing clinical investigations.

### c-ROS oncogene targeted therapies in NSCLC

*ROS1* rearrangement is characterized by 1–2% NSCLC cases, noticed more in young never smokers. The locus for *ROS1* resides on chromosome 6, encoding for a tyrosine kinase receptor with the rearrangement being exhibited by the sufferers with reasonably similar clinical symptoms to those of *ALK*-rearrangements. Crizotinib is the first distinct CD approved (March 2016) for *ROS1* mutated advanced NSCLC. Several studies evaluating crizotinib efficacy are undergoing, wherein a phase I effort revealed significant antitumor efficacy. Screening divulged a 72% ORR with 17.6 months as median duration while with mPFS and OS of 19.3 months and 51.4 months [112]. No explicit claims could be made for crizotinib antitumor efficacy as more than 80% sufferers were earlier administered at least one previous line of treatments. On similar lines, phase II crizotinib trials in Europe and East Asia, exhibited a 63% ORR with significant reproducibility and safety [112, 113]. In an open-label, single arm trial of 127 East Asian patients with ROS1 rearranged NSCLC, 71.7% ORR was noted irrespective of brain metastasis or prior treatments [114]. A low response was correlated with a baseline CNS disease, with 18.2 months PFS compared to 10.2 months in the non-CNS baseline sufferers. Importantly, 18% sufferers exhibited a baseline brain metastasis, subject to neurological stability in the last 15 days.

*ROS1* kinase abnormal NSCLC treatment has also been made with certinib, a second generation drug for the treatment of TKI-naive and crizotinib resistant ALK positive NSCLC [115]. Preclinical studies screened certinib as a selective ROS1 inhibitor with 20-fold potency over crizotinib [116–118]. A phase II clinical trial comprising 30 crizotinib naive, ROS1 positive NSCLC sufferers, demonstrated a 67% ORR with 21-month duration of response (DOR) and a 19-month mPFS [118]. Though there were 2 sufferers with a prior crizotinib therapy, no responses (NR) were observed and the trial was re-attempted on crizotinib-naive sufferers. A striking observation was the single crizotinib resistant and seven crizotinib-naive patients amongst the 8 sufferers having CNS vulnerability, with a 25% ORR and 63% disease control rate (DCR). These observations though projected certinib therapeutic utility but could not establish its efficacy in overcoming crizotinib resistance, with G2032R, D2033N, L1951R, and S1986Y/F as the troubling mutations [119–121]. Furthermore, the phase II trial on certinib revealed diarrhoea (78%), nausea (59%), anorexia (56%) and vomiting (53%) as prominent AEs to a 750 mg daily intake [111, 118, 122]. A subsequent ASCEND-8 study, however, elucidated certinib usefulness at 450 mg on daily basis, providing greater tolerance with unchanged clinical efficacy [123]. To counter the certinib inadequacy, ROS1 and ALK selective lorlatinib, was examined due to its significant CNS penetration besides the efficacy towards multiple ROS1 mutations [121, 124, 125]. In a lately single arm phase I/II trial screening 69 *ROS1* positive NSCLC sufferers (30% ROS1 TKI naive, 58% earlier treated with crizotinib and 12% treated with one, non-crizotinib ROS1 TKI or more than 2 ROS1 TKIs), an overwhelming 57% of treated individuals exhibited brain metastases at baseline with 41% ORR amongst all. Thereby, lorlatinib efficacy was noted as substantially greater in TKI naive patients (ORR: 62%, PFS: 21 months) than crizotinib (ORR: 35%, PFS: 8.5 months). Although grade 3 or 4 AEs were witnessed in 49% sufferers, no deaths occurred.

To still counter the vulnerabilities, a multikinase inhibitor, entrectinib was proposed with significant potential towards ROS1, ALK, and TRK resistance [91, 126]. This drug exhibited a better blood-brain barrier trafficking and CNS prevalence [95]. The STARTRK-1, STARTRK-2, and ALKA-372-001 trials accorded the FDA approval to entrectinib via accelerated mode for metastatic ROS1 mutant NSCLC [127, 128]. Eventually, the findings were published in 2020 [129]. The trials screened 53, ROS1 inhibitor-naive patients for ORR and DOR as primary endpoints while PFS, OS, intracranial ORR and DOR were recorded as secondary endpoints. Majority of the sufferers were females (64%), with 59% never-smokers exhibiting an ECOG (functioning ability involving self-care, daily activity and physical ability) of 51%, having received at least one prior systemic therapy (68%). With a minimal 600 mg daily (once) intake and 12 months follow-up, a 77% ORR with 24.6 months and 19 months, median DOR and mPFS, the outcomes were noted as significant. The 43% sufferers exhibiting baseline CNS had an ORR of 74% while the median DOR and PFS were 12.6 months and 13.6 months. These observations inferred a potent entrectinib efficacy, on systemic as well as in the CNS conditions. The sufferers with baseline CNS disorder exhibited an 80% ORR with 24.6 months and 26.3 months DOR and PFS. Some conclusions could not be made due to non-estimated median OS on 15.5 months follow-up. Yet again, weight gain (8%) and neutropenia (4%) were noted as significant grade 3 or 4 AE, although no deaths occurred. Readers are suggested to refer the specific source for details [130].

#### Post therapeutic mutations in ROS1 positive NSCLC sufferers

Resistant outcomes are well-known for ROS1 abnormalities, with intrinsic confrontation being the gatekeeper mutations in the ROS1 kinase region, the G2032R being the most vulnerable, forbidding the crizotinib binding [131]. Resistance in tackling ROS1-positive NSCLC is often an outcome of simultaneous *KRAS* and *MET* activation. Studies demonstrate the manifested *BRAF* and *KRAS* mutations alongside MET amp in crizotinib or lorlatinib-treated ROS1 positive NSCLC sufferers [132, 133]. While resistance to certinib and lorlatinib is attributed to their weak blood-brain access, the latest findings on entrectinib due to weak crizotinib response, surmise an important update. Reported from Japan in November 2022, the study discusses the treatment of a 74 years old, non-smoking female who was screened with stage IVB lung AC (ROS1 positivity) besides stage I right breast cancer. Five days after entrectinib treatment as a first-line NSCLC therapy, the patient developed oral dysesthesia with aggravated blood creatinine. Worsening ceased the entrectinib treatment and a follow-up of 14 days, revealed an improved status, following which entrectinib was resumed but at a reduced dose. On a 19 days observation, the patient exhibited breath shortness with bilateral lower extremity edema and respiratory failure. Laboratory screening revealed enhanced N-terminal pro-brain natriuretic peptide (NTpro BNP), troponin I, creatine kinase, C reactive protein, with trans-thoracic echocardiogram demarcated congestive heart failure. Absence of chest pain and fever resulted in concluding an entrectinib driven heart failure, the symptoms for which again improved, moderating cardiac impairment to baseline on 7 days of entrectinib termination and standard heart failure treatment. On a further dose reduction, heart failure reappeared and made the patient vulnerable to cardio-toxicity. Since crizotinib treatment remained unsuccessful, the cardio-toxicity was correlated with entrectinib intake which was subsequently withdrawn [134].

### Neurotrophic RTK

**Figure 2:**
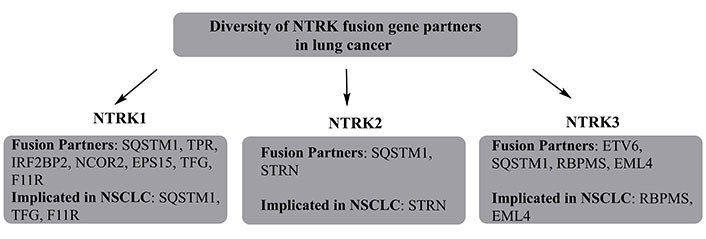
The diversity of *NTRK* fusion gene partners, with their explicit involvement in NSCLC. TPR is a tumor sensitivity index. SQSTM1: domain specific mutations in sequestosome 1; IRF2BP2: interferon regulatory factor 2 binding protein 2; TFG: tropomyosin-receptor kinase fused gene; RBPMS: RNA binding protein with multiple splicing

The *NTRK* prevails as a member of TRK family, comprising *NTRK1*, *NTRK2*, and *NTRK3* as its fusion genes encoding for TRKA, TRKB, and TRKC respectively (Figure 2). The *NTRK1* was first recognized as an oncogene in 1982 with its complementary DNA (cDNA) being isolated in 1989 [135, 136]. In 1991, TRKA expression in nervous system was unveiled, as a receptor for neutrophin nerve growth factor (NGF) with TRKB/TRKC emergence as members of the same family [137, 138].

NTRK receptors exhibit the binding susceptibility to multiple ligands, viz. NGF for NTRKA, neutrotrophin 4 (NT-4) for TRKB, and NT-3 for TRKC [139]. All known NTRKs play a decisive role in CNS functioning, including cell proliferation, migration, differentiation and apoptosis. Interestingly, the first NTRK inhibitor screening in clinical trials was not attempted till 2015, the last six years have witnessed two FDA-approved NTRK inhibitors and multiple 2nd generation drugs moving to clinical stage [140, 141]. Presently available NTRK inhibitors belong to the umbrella of TRKs, with some being active against ROS1 and ALK. These inhibitors forbid ligand-TRK interaction and concomitant TRK activation, blocking the activity of tumor cells overexpressing NTRK fusion proteins. This induces apoptosis with substantial inhibition of growing tumor cells. Two US-FDA approved NTRK inhibitors are larotectinib and entrectinib, screened via basket trials.

Larotectinib is the first approved TRK inhibitor for treating metastatic tumors harbouring *NTRK* gene fusion. In the year 2018, Drilon and team [140] published the larotrectinib efficacy in TRK fusion exhibiting tumors among the adults and children. This combined study constituted LOXO-TRK-14001 (NCT02122913), SCOUT (NCT02637687), and NAVIGATE (NCT02576431) phase I/II clinical trials. Overall, 55 patients (aged 4 months to 76 years) were screened, comprising 17 unique TRK fusion-positive tumors. The intake was optimized at 100 mg, twice daily for the sufferers having at least 1 m^2^, body surface area. For children having less than 1 m^2^ surface area, 100 mg per m^2^ dosage was optimized. Analysis revealed a 75% ORR via unbiased central review and 80% concerning the investigator’s assessment. Over a 9.4-month median follow-up, ~86% sufferers with a response progressed with the treatment, primarily having undergone curative intent intake. Major side effects included gastrointestinal effects though 30% sufferers exhibited dizziness, the gross toxicity was grade 1 and manageable. Based on these multicentred single-arm trials, the FDA sanctioned the accelerated approval to larotectinib use for adults and children, having *NTRK* gene fusion-positive solid tumors, in November 2018. Results for updated efficacy and safety vide longer follow-up, were reported in the 2021 ASCO meeting, enrolling 218 patients of which 206 were evaluable for efficacy. Amongst the screened patients, 44% were NTRK1 positive, 3% for *NTRK2* and 53% for *NTRK3*. Of the 21 distinct tumors treated, 9% belonged to the lungs. The median age was 38 years, with 45% sufferers, having received two or more, earlier systemic therapies while 27% exhibited no such history. Analysis revealed a 75% ORR with 22% complete and 53% partial respondents while 16% and 6% significant and progressive disease fates respectively. The mPFS was 35.4 months with 20.3 months follow-up. Safety evaluation on 53 patients with more than 24-month treatment did not reveal any new indications, whereby 18% of screened population had grade 3–4 AE and only 2% patients discontinued due to AEs. The observations thereby, suggested the larotrectinib medication safety [142]. The ASCO 2021 meeting updated the observations of NSCLC clinical trials NCT02576431 and NCT02122913. The entire 20 patients were heavily pre-treated with a median of 3 systemic therapies, having 48.5 years as the median age. Nineteen of screened cases had non-squamous NSCLC while one had neuroendocrine cancer, *NTRK1* gene fusion was exhibited by 80% sufferers while the remaining carried *NTRK3*. Intake was fixed at 100 mg/m^2^ continually till 28-day until the disease progression, withdrawal, or unbearable toxicity. The 15 evaluable sufferers exhibited an ORR of 73% with 3 having stable disease and one with progressive disease. The median response duration was 1.8 months while the median OS was 40.7 months at 16.2 months median follow-up. Major AEs belonged to grade 1 or 2 with no treatment discontinuation [143].

The second TRK inhibitor was entrectinib, an oral TKI for *ROS1*, *ALK*, and *NTRK* gene fusions. Inceptive approval to entrectinib was granted in June 2019 for adult and paediatric advanced NSCLC patients with NRTK fusions. In an integrated study, entrectinib displayed a 50% ORR in the NTRK fusion-positive solid tumors which improved to 70% for ROS1-positive NSCLC sufferers. Interestingly, the intracranial ORR was 55% for both *ROS1* positive and *NTRK* fusion carrying solid tumors with 12.6–24.6 month ranged median response [144, 145]. In the past three years, STARTRK-1, STARTRK-2, ALKA-372-001 (all more than 18 years with metastatic NTRK fusion-positive tumors) and STARTRK-NG (21 years, young paediatric sufferers) trials screened entrectinib anti-NSCLC efficacy [141]. In all, 54 patients having ten distinct tumors with 19 histologies, were given a minimal single entrectinib dose. With a 12.9 month follow-up, 7% sufferers exhibited a complete response (CR) while 50% responded partially. The median response was 10 months with majority of toxicities being grade 1 or 2, mainly dizziness. The most common grade 3 or 4 AE was weight gain, noticed in 10% NTRK fusion-positive safety group and 5% in the overall safe evaluable group. Relying on these observations, the US-FDA granted accelerated approval to entrectinib for adults and paediatrics, aged 12 years or more and having solid tumors characterized by an *NTRK* gene fusion with no resistance to NTRK inhibitors. Approval was also given by European Medicines Agency in 2020 and in 2021, the STARTRK-2 trial demonstrating a safety profile with least treatment burden [146].

Like other TKIs, first-generation *NTRK* inhibitors lacked the potency to counter the mutations which affected the binding interactions, including both primary and acquired resistance [147, 148]. The 2nd generation TKIs with lower molecular weights [355.37 g/moL for repotrectinib and 380.43 g/moL for selitrectinib (DS-6051b), against 428.44 g/moL and 560.65 g/moL for larotrectinib and entrectinib] and compressed macrocyclic structures, overcome these inadequacies. Their compact structure minimizes the steric hindrance which forbids the binding of 1st generation inhibitors with the ATP binding site amidst the solvent front, gatekeeper and mutations stabilizing inactive kinase conformation (*xDFG*) mutations. The compact structure of 2nd generation *NTRK* inhibitors accommodates the resistant confirmation in distinct, TRK kinase active sites. Studies probing the larotrectinib, entrectinib, selitrectinib, and repotrectinib crystal structures in the cellular modules of TRKA/B/C fusions and resistant variants, concur these findings, in xenograft tumor models [148]. Analysis revealed the highest potency for repotrectinib for TRKA/B/C fusion with < 0.2 nmol/L half maximal inhibitory concentration (IC_50_), succeeded by selitrectinib, entrectinib and larotrectinib with 1.8–3.9 nmol/L, 0.3–1.3 nmol/L and 23.5–49.4 nmol/L ranged IC_50_. Repotrectinib (TPX-0005), has lately been investigated for countering *ALK*, *ROS1* and *NTRK* mutations, exhibiting a more than 90 fold efficacy than crizotinib. Studies evaluating it in the *ROS1* positive advanced NSCLC patients elucidated its intracranial actions. A phase I trial, comprising 65 advanced NSCLC patients having *ALK*, *ROS1*, or *NTRK* positivity of which, 23 had baseline CNS abnormality. Follow-up revealed dysgeusia, dizziness, paresthesia and nausea as frequent TEAEs, dose-limiting toxicity in 2 (240 mg and 160 mg) sufferers while maximum tolerated dosage could not be reached. Significant response was observed in 8 cases, with *ROS1* and *NTRK* positivity. The ORR of 90% and 28% were noticed in 10 TKI-naive and pre-treated patients. Intake of 160 mg or more enabled a 44% response in 9 TKI pre-treated NSCLC patients, inferring below-par compatibility. Interestingly, 3 TKI naive NSCLC sufferers exhibited a 100% ORR which reduced to 50% in 4 TKI pretreated sufferers [98].

Another potent TKI having a high affinity for ROS1 and NTRK kinases is taletrectinib **(**DS-6051b), having significant tolerance besides an inhibitory response, in the phase I/Ib trial. Of the six patients screened for efficacy, two responded partially and developed a stable disease [149]. First-in-human, phase I results for taletrectinib were published in September 2020, involving 46 adult neuroendocrine sufferers exhibiting tumor-induced pain or *ROS1*/*NTRK* rearrangements. Inspection revealed nausea (47.8%), diarrhoea (43.5%) and vomiting (32.6%) as prominent AEs. Reduced pain scores were noticed following the 800 mg once-daily dose cohort. Substantiated ORR of 33.3% was noted in six patients with response evaluation criteria in solid tumors (RECIST)-evaluable crizotinib refractory *ROS1* mutated NSCLC. This study also demonstrated a prevalence of cabozantinib sensitive *ROS1* L2086F mutation as acquired resistance. The latest progress in taletrectinib evaluation is reported as a phase I study on United States and Japanaese citizens [150]. The study analysed 22 of the 61 enrolled patients, who were administered taletrectinib at 400, 600, 800, and 1,200 mg once daily via oral route. The extent was 400 mg twice daily for dose-escalation evaluation. The median follow-up time was 14.9 months, wherein 18 sufferers with *ROS1* mutation were accessible for response. To a surprise, the confirmed ORR for ROS1 TKI-naive sufferers was 66.7% with a 100% DCR while the same extent for crizotinib pretreated patients was 33.3% with 88.3% control rate. Median PFS for 11 ROS1 TKI naive patients was 29.1 months while for 8 crizotinib-refractory sufferers, it was 14.2 months. The frequent AEs noted herewith included alanine transaminase and aspartate transaminase aggravations (till 72.7%), nausea and diarrhoea (till 50%). Another potent NTRK mutation specific TKI is selitrectinib (LOXO-195), currently ongoing phase I/II study (NCT03206931, NCT03215511). Some case studies have indeed highlighted the selitrectinib potent ability. One of these is a 2021 attempt wherein, a solitary patient with mammary analogue secretory carcinoma of parotid gland and ETV6-NTRK3 fusion initially responded to entrectinib (STARTRK-2 trial). The patient subsequently developed a secondary resistance to entrectinib via *NTRK3* G623R mutation, thereafter responded to selitrectinib [151]. Another significant effort is a 2020 study, wherein a 47-year old woman carrying unclassified sarcoma and TPM3-NTRK1 fusion, initially attained a partial response (PR) towards larotrectinib. On developing *NTRK1* G595R solvent-devoid mutation, the sufferer was switched to selitrectinib, attaining PR in 3 months. Thereafter, the patient exhibited disease progression at multiple sites, which were resected via surgery. Post-surgery resumption of selitrectinib resulted in a better response and the patient lived without any disease for more than one year, herewith [152].

#### CNS metastasis restriction of first-generation NTRK inhibitors

The established significance of NTRK proteins in the CNS, the first-line NTRK inhibitors were programmed for enhanced CNS penetration. Perhaps, this was the reason that CNS activity was not screened in detail while evaluating the larotrectinib efficacy. However, analysis of entrectinib indeed involved screening the CNS action, wherein 22 of the 54 screened patients (22%) exhibited below 10% CNS metastasis with larotrectinib. The updated trial on entrectinib monitored the response of 12 sufferers, six of whom developed a PR as per a blinded unbiased review while four (33%) sufferers developed a stable disease [141]. The time for CNS progression was 17 months. The initial larotrectinib trial involved merely nine sufferers having primary CNS tumors. But the extended study which got published in 2021, 19 of the 218 (8.7%) sufferers had brain metastasis at baseline of which 15 remained available for assessment, exhibiting a 73% ORR for the brain metastases [142]. The extended study of larotrectinib in *NTRK* fusion harbouring NSCLC sufferers comprised 8 evaluable patients for assessable CNS metastasis, who developed a 63% ORR [142]. Though this trial did not rigorously screen the intracranial ORR, the CNS progression seemed unlikely, with larctrectinib. Even though published studies screen the CNS activity more critically for entrectinib, the response monitoring inevitably concluded both larrotrectinib and entrectinib as potent guards against CNS metastasis of tumors.

#### Resistance to NTRK inhibitor based therapies

**Figure 3:**
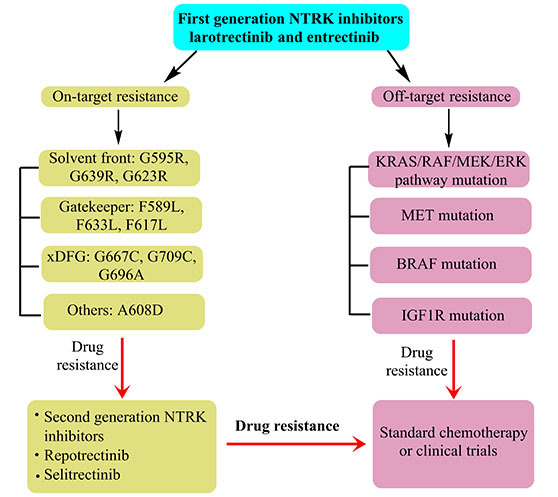
Resistant response manifestations of NTRK-inhibitors in the NSCLC treatment

Resistant responses are quite frequent for the first and second-generation NTRK inhibitors. Reduced potency of first generation NTRK inhibitors was inferred from their weaker actions on TRK mutations, including those of solvent front TRKA G595R, TRKB G639R, TRKC G623R, TRKC G623E; gatekeeper TRKA F589L, TRKB F623L, TRKC F617I; xDFG TRKA G667C, TRKB G709C, TRKC G696C; and compound mutation TRKA G595R, RKA F589L. The NTRK drug resistance could be on-target (via mutation of TRK kinase domain) or off-target (bypass or downstream pathways) (Figure 3) [96, 147, 148]. The former of these responses could occur in the TRK kinase domain via mutations in the solvent front domains (TRKAG595R, G667C, TRKBG639R, TRKCG632R), gatekeeper locus (TRKAF589L), or kinase activation loop xDFG location (TRKAG667S or TRKCG696A) [153]. Usually, on-target resistance should be confronted with next-generation *NTRK* inhibitors while for acquired NTRK resistance; clinical attempts reported a better response with repotrectinib.

Some reports do reveal an inadequate efficacy of 2nd generation NTRK inhibitors towards off-target acquired resistance. As of now, most observations are the outcomes of clinical case reports. Another important scenario pertains to the fact that most of the resistant responses are the outcomes of sequential treatments with second-generation *NTRK* inhibitors. Thereby, a significant possibility is there that the listed resistant responses are manifested towards both first and second-generation *NTRK* inhibitors. Replete evidence from gathered studies lists RAS/RAF/MEK/ERK pathway as the underlying signaling network. For instance, one sufferer with PLEKHA6-NTRK1 fusion-positive cholangiocarcinoma, acquired resistance to entrectinib because of the aggressive *MET* focal amp wherein no new, on-traget resistance could be screened. As *MET* aggravation contributed to TRK-independent resistance, the sufferer did not respond to selitrectinib. Eventually, crizotinib (a *MET* inhibitor) treatment resulted in significant betterment. Quite unexpectedly, the post-progression analysis of prenatal cell free DNA revealed overexpressed focal *MET* besides 13 emergent missense mutations, most of which inhibit crizotinib binding. This implied a prevalence of multiple resistance mechanisms, drawing support from the animal model and cell line studies. For instance, expression of TPR-NTRK1 fusion kinase in immortal mouse lung epithelial cells, fuelled the tumor growth [154], the same cells exhibited a dramatic suppression of *in vivo* drug resistance on being administered entrectinib + cobimetinib (a MEK1/2 inhibitor). Thereby, the logic of simultaneous TRKA and MEK1/2 inhibition in NTRK1-driven tumors is validated. Interesting findings in pancreatic cancers (pancreas) demonstrated cross-resistant outcomes, for *ETV6-NTRK3* and *TPR-NTRK1* fusion gene positivity, developing resistance towards selitrectinib (MEK1 P124S mutation) and entrectinib (ErbB2 S310F mutation) [155]. Recent findings in particular, infer an increasing reliance on screening individual resistance mechanisms and targeting before proceeding with combinatorial therapy-mediated treatment of multiple resistance pathways. A decisive factor affecting the success of such a regimen is the accurate identification of a dominant resistance mechanism, in the absence of which the toxicity aspects indeed prelude to a sharp concern. In the event of non-traceable targetable mutations, standard chemotherapy with immunotherapy is the inevitable recourse. Rapid resistant observations imply a rather inevitable need to adopt NGS on tumor biopsy for stable conditioning.

### MET factor

The noted proto-oncogene, *MET* resides on chromosome 7q21–q31 and encodes for a transmembrane receptor, referred as “hepatocyte growth factor receptor”. A tyrosine kinase receptor that binds with hepatocyte growth factor (HGF), the *MET* gene regulates cell proliferation, survival, migration, invasion, angiogenesis and epithelial to mesenchymal transition via downstream activation of RAS/ERK/MAPK, PI3K-AKT, Wnt/β-catenin, STAT signaling pathways [156]. *MET* dysregulation involves sequential heterogeneous alterations, including amps and exon 14 abnormalities, extending the activation of cellular MET receptor and downstream signaling pathways [46]. Exon 14 skipping mutation (EM) of *MET* has been demonstrated as a discrete NSCLC hallmark, prevailing discretely from the *EGFR*, *ALK* and *c-ROS* mutations besides being associated with a poor prognosis. Several studies describe MET exon 14 EMs as clinically distinct NSCLC subtype, addressed via patient stratification-driven personalized therapy [157, 158].

Abnormalities in *MET* gene expression are noticed in less than 5% of NSCLC sufferers. Usually, MET amp is ascertained via fluorescence *in situ* hybridization (FISH) and next generation DNA or RNA sequencing. The last two decades have witnessed multiple MET targeting agents comprising multikinase inhibitors (crizotinib, cabozantinib, MGCD265, AMG208, altiratinib, golvatinib), selective inhibitors (capmatinib, tepotinib, tivantinib) and monoclonal antibodies (mAbs; onartuzumab, emibetuzumab, ficlatuzumab, rilotumumab). Crizotinib, cabozantinib, tepotinib, capmatinib, amivantamab and savolitinib are the noted CDs evaluated for *MET* mutated anti-NSCLC therapy [159, 160]. Crizotinib is a multi-targeted small-molecule TKI, majorly targeting the *ALK*, *ROS1*, and *MET* genes. Clinical potency of crizotinib against MET exon 14 skipping harboring NSCLC is notified in selective cases. A phase I PROFILE 1001 study on 69 sufferers was attempted by Shaw and teammamtes [112], wherein crizotinib treatment (as singular agent) was screened for 7.3 months (mPFS), reaching a 32% ORR. Tepotinib prevails as an exclusive MET selective inhibitor, which disrupts the MET signal transduction for its potent anti-NSCLC activity [161]. Screening the tepotinib efficacy in exon 14 altered NSCLC patients, a phase II trial (VISION) by Paik and associates [162] observed a significant response via 500 mg daily administration in 152 patients. Follow-up of 99 of these sufferers till a minimum of nine months, revealed tepotinib association with PR in ~50% sufferers, with a 46% ORR (95% confidence), monitored via an independent review and 56% investigator assessment. The PFS and OS were 8.5 months and 17.1 months. The findings awarded regulatory approval to tepotinib in *MET* exon 14 EMs, in March 2020 in Japan [162].

Capmatinib is another selective MET inhibitor, which impairs the downstream effector’s activation in the MET signaling pathway by hindering MET phosphorylation. Screening the capamitinab efficacy via a phase II study, involving *MET* exon 14 skipping NSCLC patients (assigned to cohorts as per the previous lines of therapy), Wolf and associates [163] examined the sufferers having received one or two lines of therapy. The investigators noted that a 400 mg capmatinib tablet intake twice daily, exhibited a 41% ORR and a mean PFS of 52 months. The extents were 68% (ORR) and 12.4 months (PFS) in treatment naive sufferers [163]. Yet another phase II study screened capmatinib efficacy in 97 sufferers via post-therapy screening and noticed an ORR of 31.9% in pretreated patients whereas treatment naive sufferers exhibited a response of 71.4%. Familiar AEs in both categories were peripheral edema, nausea, and vomiting [164]. Savolitinib is a *MET* receptor inhibitor that specifically inhibits *MET* activation by disrupting the *MET* signal transduction in an adenosine triphosphate competitive regime, whereby it inhibits the tumor cell growth [165]. In a recent trend, Lu and accomplices [166] performed a multicenter phase II study to screen the savolitinib efficacy, corresponding to 600 mg and 400 mg in Chinese NSCLC (exon 14 altered) patients. Twenty-five of the examined sufferers had pulmonary sarcomatoid carcinoma (PSC), a rare aggressive form of NSCLC while 45 others exhibited NSCLC histology. The primary endpoint as ORR was monitored in tumor response evaluable (61 patients) while the sensitivity screening was done in all screened 70 patients. Sufferers of both groups exhibited significant ORR (49% in an evaluable group having *MET* exon 14 mutated NSCLC and 42.9% in the full analysis set), both with 95% confidence limits. Savoliyinib provided significant survival betterment irrespective of tumor subtypes with more than 40% ORR of each group as well as in treatment-naive patients. Significantly though, savolitinib also proved significant in arresting brain metastasis of tumors [166]. The studies discussed, thereby suggest a superior efficacy of *MET* selective TKIs to treat exon 14 skipping mutated NSCLCs than the non-selective TKIs. It summarizes the clinical trials evaluating the MET inhibitors specifically targeting the exon 14 EM for NSCLC manifestation (Table 3) [162, 163, 166, 167].

**Table 3:** Summary of clinical trial outcomes on some MET inhibitors, specifically targeting exon 14 EMs

**Study design**	**Cancer type and study population (MET positive)**	**MET alteration type & therapy**	**ORR (%)**	**mPFS and OS (months)**
Phase I (NCT00585195) PROFILE 1001	NSCLC, *n* = 69 (65 evaluable)	*MET* exon 14 alteration Crizotinib 250 mg BID in continuous 28-day cycles	32 (95% CI)	7.3 (95% CI, 5.4–9.1) and 20.5 (95% CI, 14.3–21.8)
Phase II (NCT02864992) VISION study	NSCLC (advanced/metastatic), *n* = 169 (152 received treatment)	*MET* exon 14 alteration SM Tepotinib 500 mg OD	Independent review 46%; investigator assessment 56%	17.1
Phase II (NCT02414139)	NSCLC (stage IIIB/IV), *n* = 97 (cohort 4:69 points; cohort 5:28 points)	*MET* exon 14 alteration SM Capmatinib 400 mg BID	Cohort 4: 41%; cohort 5b: 68%	NR
Phase II, (NCT02897479)	PSC; NSCLC, *n* = 593 [70 (60 evaluable: 25 PSC, 45 other NSCLC)]	*MET* exon 14 alteration SM Savolitinib (oral intake) 600 mg (for weight more or equal to 50 kg) and 400 mg (for weight less than 50 kg) until disease progression or intolerable toxicity OD	Tumor response evaluable set: 49.2 (95% CI, 36.1–62.3); FAS 42.9 (95% CI, 31.1–55.3)	12.5 (95% CI, 10.5–23.6)

BID: twice a day; OD: once a day; CI: confidence interval; *n*: number of people

Amivantamab is a prominent antibody, known for its *EGFR* and *MET*-targeting specific actions [168]. Multiple *in vitro* and *in vivo* studies have demonstrated the amivantamab’s ability to inhibit the EGFR and MET signaling via ligand blocking and biochemical degradation. Noted amivantamab actions, include its ability to induce trogocytosis and allocate immune effector cells for eliminating EGFR and MET expressing tumor cells, *vis-à-vis* antibody-dependent cellular cytotoxicity [168, 169]. In a phase I study involving metastatic NSCLC, amivantamab was screened for varied tumor subgroups, comprising *EGFR* exon 20 insertions, *MET* exon 14 and *MET* amp mutations. Though MET mutation-specific results are not yet reported, the 40% ORR towards *EGFR* exon 20 insertions led to the amivantamab FDA approval for *EGFR* exon 20 insertions in NSCLC patients after platinum chemotherapy progression [169]. It would be significant to screen the efficacy of remaining cohorts, wherein amivantamab expansion to counter MET exon 14 mutation and *MET* amp NSCLC could be validated.

#### MET targeted therapies mediated confrontation of EGFR resistance in NSCLC

Crosstalk of MET and EGFR signaling pathways suppresses the TKI-targeted therapies, as evidenced by a correlation of *EGFR* and *MET* activations [170, 171]. Aggravated MET signaling stimulates downstream signal transduction evading tumor cell death by EGFR-TKIs. MET signaling thereby, contributes to mounting EGFR-TKI resistance, exhibiting a 15% probability after first-line treatment, enhancing the osimertinib resistance via 19% reduced efficacy [172, 173]. It is pertinent to mention here that relative extent of MET activation could significantly differ for the EGFR-TKI therapies administered and treatment naive NSCLC sufferers. Thereby, using MET inhibitors in NSCLC sufferers with acquired resistance to EGFR-TKIs and treatment-naive patients, inevitably exhibits distinct feasibilities [174]. The successful outcomes of anti-MET and anti-EGFR drugs infers a *MET-EGFR* heterogeneity impaired success of anti-NSCLC therapies (Table 4) [175–180].

**Table 4:** Combinatorial therapies comprising of anti-MET and anti-EGFR programmed drugs for advanced NSCLC treatment

**Combination studied**	**Optimizations of clinical trails**	**Key findings**	**Results/Generalizations affirmed**
Tepotinib and gefitinib	A single phase Ib/II multicenter randomized trial, screened patients were *EGFR* mutant sufferers with overexpressed *MET*. Phase II study examined 55 patients, of which 31 received 500 mg tepotinib and 250 mg gefitinib daily. Twenty four patients received pemetrexed (500 mg/m^2^) + cisplatin (75 mg/m^2^) or carboplatin	Enhanced survival for tepotinib + gefitinib co-administered patients, exhibiting 37.3 months OS and 8.3 months PFS contrary to 17.9 months and 4.4 months for chemotherapy administered sufferers	Anti-EGFR and Anti-MET combinatorial therapies exhibited a better potential than standard chemotherapy in patients harbouring acquired resistance to EGFR inhibition
Osimertinib and savolitinib	Examined patients prevailed in B and D expansion cohorts, group B comprised 3 sub-cohorts (B1, B2 and B3). B1 included (*n* = 69) patients pretreated with osimertinib. Sub-cohorts B2 and B3 included patients with T790M positivity (*n* = 18) and negativity (*n* = 51) but no prior osimertinib treatment. Cohort D included *MET* overexpressing and *EGFR* mutant sufferers, earlier treated with 1st or 2nd generation EGFR-TKIs, all patients in this group were T790M negative	Sub-cohorts B and D responded better with 67%, 64% and 11 months, 9.1 months ORR and mPFS, contrary to those who received an earlier treatment of 3rd generation EGFR-TKI	Osimertinib + savolitinib combination responded significantly in NSCLC sufferers harboring MET initiated resistant response towards EGFR-TKIs
Capmatinib and gefitinib	Patients exhibiting *MET* amp and mutant *EGFR* expression were examined in a a phase Ib/II settings, with failure of EGFR inhibiting therapy. Sample size comprised 100 patients who were administered 400 mg capmatinib + 250 mg gefitinib	Screening revealed 29% ORR with 5.5 months PFS for capmatinib + gefitinib. Subgroup screening revealed a better response for more than 6 *MET* gene copy number (GCN) with an ORR of 47%	Results established the capmatinib and gefitinib combination as highly feasible and promising treatment option for *EGFR*-mutated, *MET* dysregulated NSCLC and morespecifically for those having *MET*-amplified tumors
Combinations of small molecule inhibitor and mAb (capmatinib with gefitinib, telisotuzumab with erlotinib, savolitinib with gefitinib, onartuzumab with erlotinib, capmatinib with erlotinib and emibetuzumab with erlotinib)	Chosen patients were all *MET* mutant, a noted attempt (phase II study) by Camidge and colleagues [180] co-delivered emibetuzumab (750 mg, every 2 weeks) with erlotinib (150 mg, OD) intravenously and compared the outcome with emibetuzumab monotherapy (750 mg, intravenous) in the sufferers with acquired resistance to erlotinib and MET positive sufferers	All combinations revealed a better response with 3.3–5.6 months PFS In the study by Camidge and colleagues [180], emibetuzumab + erlotinib responded better with 3.3 months PFS and 3% PRR contrary to the 1.6 months PFS and 4.3% ORR for emibetuzumab alone	Emibetuzumab-erlotinib combination is more effective to treat *MET* mutated NSCLC than the singular treatment with emibetuzumab

PRR: pattern recognition receptor

#### MET targeted therapies in NSCLC with *de novo* amp

Unlike the discussed gene mutations in NSCLC, tumors exhibiting potentially newer amplified *MET* overexpression have been screened as exclusively MET signaling dependent. These abnormal *MET* expressions [chromosome 7 centromere (CEP7) ratio ≥ 5] have been screened in < 5% NSCLCs and frequently contribute to abysmal prognosis [181]. The success of anti-MET targeted therapies for NSCLC treatment is curtailed by two major factors, the first of which necessitates a threshold *MET* amp. Multiple evidences correlate a high level *MET* amp as more reliable indicator of oncogenic *MET* involvement, which could further be optimized as targeted therapies. Accompanying paradox is the fact that *MET* overexpression is a poor predictor of MET TKIs efficacy without known instigators of *MET* dependence. A summary of clinical trials using MET inhibitors to monitor its amp is thereby discussed ahead.

The PROFILE 1001 study by Camidge and colleagues [182] monitored the crizotinib actions in *MET*-overexpressed NSCLC sufferers and noticed a 40% ORR for the patients with ≥ 4 *MET* to CEP7 ratios. The corresponding ORR extents for (1.8–2.2%), low and (2.2–5%), medium *MET* to CEP7 stoichiometries were 33.3% and 14.3%, suggesting a clear benefit of MET targeted therapies on sufferers having high-level *MET* amp [182]. The GEOMETRY trial screened the capmatinib efficacy and safety in high level *MET* amplified NSCLC patients (GCN ≥ 10) contrary to < 4 (low level) and 4–9 (mid-level) extents. Analysis revealed a 40% ORR with 4.2 months PFS for sufferers with GCN ≥ 10, suggesting a better response for higher *MET* expression [163]. Recent studies have demonstrated the accuracy of several novel *MET* inhibitors, including Sym 015, JNJ-372, ningetinib, bozitinib, telisotuzumab vedotin and TR1801-ADC, currently undergoing clinical investigations.

### Rearrangement during transfection

Rearrangement during transfection (*RET*) gene exerts via RTK, a proto-oncogene located on chromosome 10 (10q11.2), encoding for RET protein. *RET* rearrangements account for 1–2% NSCLC adversities (majorly LUAD) and prevail in a mutually exclusive regime to *EGFR*, *ALK*, *ROS1*, and *KRAS* mutations [183]. To date, most frequent partner of RET rearrangements in NSCLC is kinesin family member 5B (KIF5B), prevailing as the KIF5B-RET fusion gene, with a higher frequency in young non-smokers [184, 185]. Selpercatinib is the first FDA-approved *RET* inhibitor for NSCLC treatment [186]. Bevacizumab [targets vascular endothelial growth factor (VEGF) in advanced non-squamous NSCLC] and several multi-kinase inhibitors (cabozantinib, vandetanib, lenvatinib and sunitinib) are being evaluated for their anti-VEGF activities. The VEGF is critically implicated in angiogenesis which further aggravates the metastasis and tumor cell invasion. Cabozantinib and vandetanib are the prominently screened *RET* inhibitors, wherein a 20–50% response has been observed in the *RET* rearranged NSCLC sufferers [187, 188]. With a 16–20% response rate, lenvatinib and sunitinib occupy the second place but both amiss the RET targeting activity [189, 190].

Insignificant actions of multi-targeted TKIs in RET-rearrangements characterized NSCLC, have gradually switched the attention to the next generation, RET-selective inhibitors, such as RXDX-105, BLU-667, and LOXO-292. RXDX-105 is a VEGF receptor (VEGFR)-sparing RET inhibitor, with a phase Ib study on 21 untreated RET fusion-positive NSCLC sufferers, observed response in 6 of the 8, non-*KIF5B-RET* fusion positive patients. Unfortunately, none of the 13 patients carrying *KIF5B-RET* fusions met the prescribed response evaluation criteria for solid tumors [191]. Evaluation of BLU-667 exhibited a ten-fold higher efficacy than FDA approved anti-RET (oncogenic rearrangements) MKIs. Investigation via phase I study revealed an objective response in 50% (7 of the 14) BLU-667 administered RET-fusion carrying NSCLC sufferers [192]. In a more recent attempt, 79 advanced LC subjects harboring *RET* fusion rearrangements were administered BLU667. Analysis revealed a 56% ORR in 57 screened patients, with a 91% DCR. Six of the screened patients exhibited a > 6-month response with hypertension, constipation, increased aspartate aminotransferase (AST) and alanine aminotransferase (ALT), and reduced neutrophil count, as major TEAEs [193]. LOXO-292 is another RET-selective TKI, whose efficacy towards RET-driven cancers, was evaluated via phase I basket trial, at the American Society of Clinical Oncology Annual Meeting in 2018. In all, 27 NSCLC patients were recruited, 17 of which attained an evaluable ORR (one withdrew), with a predicted response time of > 6 months [194]. Of note, each of the early phase trials (on RXDX-105, BLU-667 & LOXO-292 exhibited the CNS penetration to a certain extent.

A phase I/II, ARROW, multi-cohort study evaluated pralsetinib on *RET* fusion-positive 281 NSCLC patients, aged > 18 years. Yester success via the oral route apart from CNS penetration, selective *RET* inhibition, and clinical efficacy in *RET* fusion harboring NSCLC sufferers were the encouragements to pursue the studies. Inspection revealed an ORR of 72% in treatment naive patients while for prior platinum chemotherapy administered sufferers, the extent was 59% (both estimates made at 95% CI). While the median response duration for treatment naive sufferers could not be reached, it was 22.3 months for platinum chemotherapy-administered patients. Inspection revealed tumor shrinkage in all treatment naive sufferers while in platinum chemotherapy-administered patients, the extent was 97%; the mPFS was 13 months and 16.5 months respectively. The intracranial response rate in the sufferers with measurable intracranial metastasis was 70%, with all having received prior systemic treatment. Noted AEs in treatment naive sufferers included neutropenia (18%), hypertension (10%), increased blood creatine phosphokinase and lymphopenia (9% each). The findings validated pralsetinib efficacy with tolerable response in treatment-naive, *RET* fusion-positive advanced NSCLC sufferers. Phase III trial (NCT04222972), evaluating pralsetinib and standard approach in first-line setting is currently awaiting a response [195].

### BRAF V600E mutations

This mutation is noticed in 1–3% NSCLC patients with a history of smoking and the altered *BRAF* gene, forms a non-native BRAF protein that fuels the tumor cell growth. Dabrafenib (Tafinlar) and trametinib (Mekinist), respectively the BRAF and MEK inhibitors, are used to ramify these mutations. A typical dabrafenib and trametinib combination was approved for the treatment of *BRAF* V600E mutation exhibiting NSCLC, based on the results of an open-label trial. Of the 93 enrolled patients, 57 had received a prior systemic treatment while 36 were naive. Each patient received 150 mg dabrafenib orally twice (on a daily mode) and 2 mg trametinib, OD. Analysis revealed 63% and 61% ORR in pretreated and treatment naive sufferers. The corresponding response duration was ≥ 6 months for 64% previously treated and 59% treatment naive sufferers [196, 197].

## Immunotherapy

**Figure 4:**
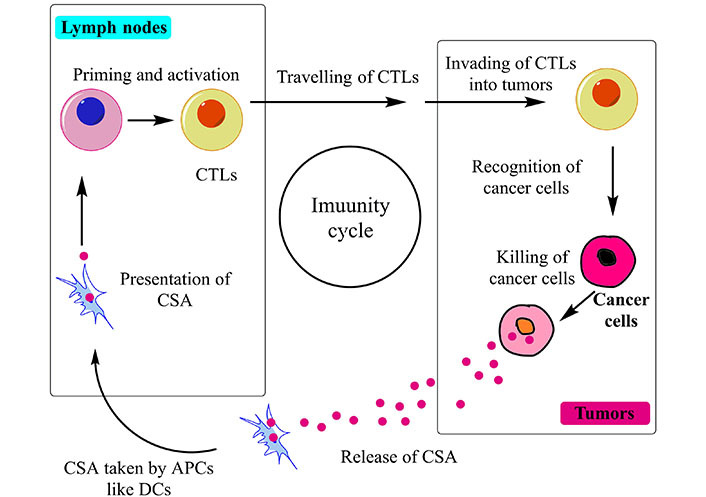
Schematic representation of cancer immunity cycle, the functioning is based on the accurate identification of CSAs by T_c_

**Figure 5:**
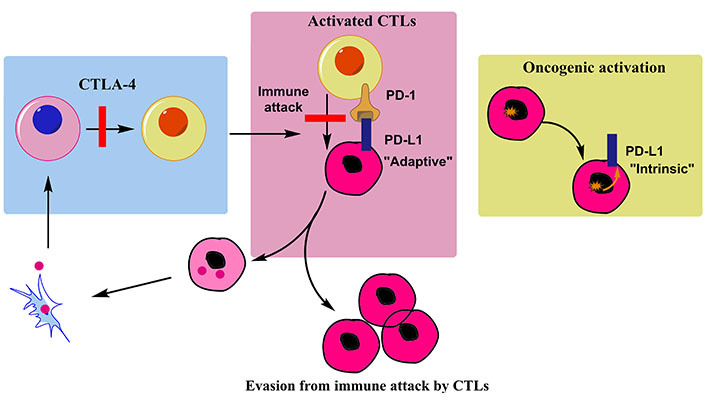
Working mechanism of immune cells involved in immunotherapy mediated treatment of tumor cells. Binding of precursors with their ligands (like PD-L1 with PD-1) inhibits their interaction with tumor cells. Therapies are therefore programmed to inhibit this ligand binding with precursor cells

One of the most recent therapies for metastatic NSCLC is immunotherapy. It is distinct from chemotherapy, as it utilizes the immune system to kill cancer and stop cancer from spreading rather than attacking cells as they divide. The immune system’s capacity to restrain itself from attacking healthy body cells is a crucial component. The immune system can halt the growth and spread of malignant tumors in the human body by the removal of abnormal cells via the cytotoxic T lymphocytes (CTLs). The process by which an anti-cancer immune response is produced is known as the “immunity cycle” (Figure 4). First, antigen-presenting cells (APCs) like dendritic cells (DCs) phagocytose cancer-specific antigens (CSAs) derived from tumor cells, which are then processed to the peptides [198]. Secondly, the DCs present the antigens on the surface of major histocompatibility complex (MHC) class II molecules to the CD4^+^ cells, generating the effector T lymphocytes against CSAs. Subsequently, the activated effector T cells travel through the blood vessel and invade the tumor area. Last but not least, CD8^+^ CTLs (T_c_) recognize the tumor-specific antigen via interaction with T cell receptor (TCR) and the CSA presented on MHC-I cells, and then attack and destroy tumor cells via perforin and granzyme release (Figure 5). To initiate an immune response, “checkpoint” proteins on immune cells act as switches that must be activated or inactivated [199]. These checkpoints are occasionally used by cancer cells to escape immunosurveillance. Some NSCLC patients are treated with medications that target these checkpoints, referred to as immune checkpoint inhibitors (ICIs).

Heterogeneity and highly random mutations in targeted proteins seem as the major constraint limiting the long-term success of chemotherapy. Besides, the functional diversity of targeted molecular aberrations is enormously greater than the one in immunotherapy. Studies that characterize simultaneous mutations of two or more genes in NSCLC therapies provide stout evidence for this. Each new variation of this kind fails to fit well with the programmed tuning of CDs, leading to a resistant response. Perhaps this seems a big reason why chemotherapy alone attains an early saturation in the treatment as compared to combinatorial mode. Although not in entirety, this issue is much lowly encountered during immunotherapy as it has comparatively fewer targets, which helps to control the corresponding functional diversity. The following paragraphs briefly describe various inhibitors that are encountered by the tumor/cancer cells and prevent them to escape from immunosurveillance.

### Targeting immune checkpoints

Immune checkpoints are the co-stimulatory and co-inhibitory receptors that help in the priming and activation of T cells. Most extensively studied immune checkpoint receptors for cancer therapy are cytotoxic T-lymphocyte-associated antigen 4 (CTLA-4) and PD-1. PD-1 is an immunological receptor from the CD28/CTLA-4 family that inhibits the antigen receptor signaling via localizing the protein tyrosine phosphatase, SHP2 on interacting with its ligands, PD-L1 or PD-L2. Owing to significant ligand diversity, this receptor is involved in manifold immune responses.

Therapies targeting PD-1 are programmed to inhibit the ligand binding with the receptor (PD-L1 inhibitors), generating the requisite immune response against the tumor cells [200, 201]. Numerous cells express ligands that bind these receptors and block the immune response to self-antigens under apt physiological circumstances [202]. Many tumor cells use this mechanism to evade immunosurveillance by ligand bound state of these receptors [203]. By obstructing these inhibitory pathways, the ICIs activate the T-cells against tumor cells to kill the tumor cells [204]. Antibodies targeting immune checkpoints result in enhanced immunologically triggered tumor cell death. Following paragraphs briefly summarize the recent studies using CTLA-4 and PD-L1 inhibitors for NSCLC treatment.

#### Inhibitors of CTLA-4

Ipilimumab was the first fully human immunoglobulin G1 (IgG1) kappa antibody ICI approved by FDA as an anticancer drug in March 2011. CTLA-4 (CD152) is a B7/CD28 family member that expresses on T lymphocytes after activation and blocks T cell functions. It competes with CD28, a co-stimulatory receptor on T lymphocytes, by binding with B7 (CD80 or CD86) on APCs with greater affinity. When CTLA-4 binds to B7, an inhibitory signal is produced [205]. One such ICI is ipilimumab that targets CTLA-4 inhibitory receptor and exhibits promising outcomes for treating last-stage melanoma [206].

In a phase II study, 53 of the 44 eligible patients were randomly administered nivolumab (23 only) while 21 were administered nivolumab + ipilimumab. These treatments succeeded by surgery in 44 candidates with operable NSCLC, using major and complete pathologic response [major pathologic response (MPR) and complete pathologic response (CPR)] as the primary endpoint. Nivolumab monotherapy resulted in an MPR of 24% (5/21) among the 37 resected patients, whereas the MPR was 50% (8/16) for nivolumab + ipilimumab treatment. Nivolumab alone had a 10% (2/21) CPR while dual therapy had a 38% (6/16) CPR. Further, inspection revealed that nivolumab + ipilimumab-treated specimens had fewer tumor cells than nivolumab-treated tumors. In the tumors treated with nivolumab alone, no significant changes in immune cell sub-populations were noticed from pre- to post-therapy. On the contrary, changes in CD3^+^ T lymphocytes from pre- to post-therapy were significantly higher in tumors treated with nivolumab + ipilimumab [207].

#### PD protein and its ligands (PD-1/PD-L1)

At present, nivolumab, pembrolizumab, atezolizumab, durvalumab and avelumab are the noted FDA-approved PD-L1 inhibitors, used in NSCLC treatment. A schematic listing of PD-1/PD-L1 therapies with the optimized dosage regimen and clinical progress, using these drugs is compiled in Table 5. Several studies have used these molecules in varied stoichiometry with CDs that have inevitably resulted in enhanced tumor cell damage [208–223]. The tangential functional aspects of these molecules are discussed in the following paragraphs.

**Table 5:** Summary of potential anti-PD-L1 monoclonal antibodies, describing their clinical trials, FDA approval, and recommended dosages [208–223]

**PD-L1 inhibitor**	**Description**	**Salient clinical trials summary**	**Results of the observations**
Nivolumab	An IgG4 fully human mAb which functions against PD-1 by disrupting its binding with PD-L1 & the henceforth downstream signaling that inhibits the antitumor T-cells	Two phase III clinical trials, CHECKMATE 017 and CHECKMATE 057 on patients who had progressed after platinum-based doublet chemotherapy Not suitable for PS of 2 or symptomatic brain metastasis	FDA approval in October 2015 for treating metastatic patients on or after platinum chemotherapy. Recommended dose: 3 mg/kg intravenously every 2 weeks.
Pembrolizumab	An IgG4 humanized antibody directed against PD-1	Screened as second-line NSCLC treatment (KEYNOTE-001) One phase II/II (KEYNOTE-010) and the second phase III (KEYNOTE-024) trial	KEYNOTE-001: accelerated FDA approval in October 2015 for tumors expressing PD-L1 and a disease progression on or after platinum chemotherapy. Other trials conferred FDA approval in October 2016 for first-line treatment of metastatic patients having 50% or more cells expressing PD-L1 with no EGFR or ALK aberrations & prior systemic chemotherapy. Recommended dose: 200 mg intravenously every 3 weeks.
Atezolizumab	A human IgG1 mAb which targets the protein PD-L1	Phase II (POPLAR) and a phase III (OAK) trials A phase II (BIRCH: exploring the efficacy as 1st, 2nd and 3rd line drug after platinum therapy), phase III (Impower-150: combined delivery with carboplatin, paclitaxel, and bavacizumab) and a phase II (CITYSCAPE: combined delivery with tiragolumab)	POPLAR and OAK trials conferred FDA approval in October 2016 for treating patients having disease progressed on or after platinum chemotherapy. Recommended dose: 1,200 mg intravenously every 3 weeks. Other trials ensured a first-line usage as monotherapy or in combination with immunotherapy.
Durvalumab	A fully human IgG1 mAb binds PD-L1 on tumor cells	A phase II (ATLANTIC, 44 patients, administered after two rounds of chemotherapy) and a phase III PACIFIC (713 patients with stage III disease, administered after two or more cycles of platinum chemotherapy)	FDA approval in February 2018 for treating unresectable stage III tumors that have not progressed following platinum chemotherapy and radiation therapy. Recommended dose: 10 mg/kg intravenously every 2 weeks.
Avelumab	An entirely human IgG1 mAb that targets PD-L1 possesses a characteristic antibody-dependent cell-mediated cytotoxicity which is missing in other PD-L1 inhibitors	Phase 1b (JAVELIN, 184 stage IIIB or IV patients progressive on platinum chemotherapy) and phase III (JAVELIN, 792 stage IIIB or IV sufferers exhibiting disease progression on platinum chemotherapy were randomly administered avelumab every 2 weeks or docetaxel every 3 weeks	Phase 1b trial achieved 50% disease control as the best response. Phase III trial revealed a better safety profile in avelumab-administered sufferers (64%) against the 86% for docetaxel. The trial is still ongoing but enrolment is closed.

PS: performace status

##### Nivolumab

Nivolumab is a fully human IgG4 antibody targeting PD-1, administered intravenously, with a mean half-life time (t_1/2_) of 25 days and it was the first ICI approved by the U.S. FDA to treat advanced NSCLC patients [224]. This drug prevents the interaction of PD-1 with PD-L1/PD-L2 and inhibits T-cell growth, gradually converting them to T_reg_ cells and suppressing the anti-tumor T-cell activity [225]. In phase I and II trials, nivolumab revealed the ~15% and ~17% efficacy on 8 months to 9 months with 41% and 19% median OS at 1 year and 3 years in the treated advanced squamous NSCLC patients [226, 227]. Readers are suggested to refer Table 4 for the nivolumab clinical trials.

##### Pembrolizumab

This anti-PD-L1 drug is an IgG4 humanized antibody, having been screened via KEYNOTE-001, KEYNOTE-010, and KEYNOTE-024 clinical trials (Table 4). Apart from these, a phase II/III trial reported 10.4 months and 12.7 months as median OS with 2 mg/kg and 10 mg/kg dosages respectively, longer than docetaxel (8.5 months). The same extents however revealed median OS of 14.9 months and 17.3 months in patients having at least 50% tumor cells expressing PD-L1 than docetaxel (8.2 months). Though no significant differences were observed for pembrolizumab and docetaxel, 2 mg/kg and 10 mg/kg pembrolizumab prolonged the mPFS by 5.0 months and 5.2 months, respectively [212].

##### Atezolizumab

Atezolizumab is a humanized IgG1 MAb targeting PD-L1 with a t_1/2_ of 27 days. Normally, PD-L1 inhibitors target the ligands on tumor cells, but atezolizumab targets the PD-L1 protein. Initial attempts establishing atezolimuab success included phase II (POPLAR) and phase III (OAK) trials, fetching it the FDA approval in October 2016 for NSCLC sufferers having progressive disease after platinum chemotherapy. Subsequent phase II, BIRCH, and CITYSCAPE trials screened the atezolizumab efficacy in singular and combinatorial mode, as first, second, and third-line drug (Table 4). While further studies continue, atezolizumab first-line use is proven with significant reliability.

##### Durvalumab

Durvalumab is a fully human IgG1-MAb that binds PD-L1 on tumor cells and has a t_1/2_ of 18 days. Noted studies probing durvalumab anti-NSCLC efficacy are phase II (ATLANTIC) and phase III (PACIFIC) trials, the latter conferring it the FDA approval in February 2018 (Table 4). The recommendation was made for patients with no disease progression subsequent to platinum chemotherapy and radiation therapy. Durvalumab efficacy at 10 mg/kg inferred its greater toxicity than pembrolizumab and atezolizumab.

##### Avelumab

Avelumab is yet another fully human IgG1-MAb that targets PD-L1 and exhibits a distinct antibody-dependent cell-mediated cytotoxicity. The drug lyses targeted tumor cells with the assistance of peripheral blood mononuclear cells and natural killer (NK) cells besides an inherent anti-PD-L1 activity [223].

### Cytokine immunotherapy

Cytokines are the small secretory proteins produced by immune (majorly) and non-immune cells, serving as molecular messengers for interacting with other cells. Their decisive roles in tumor aggravation are characterized by regulatory participation in cancer immunity cycle, including cancer antigen presentation, T cell priming and activation, effector T cell infiltration to the tumor location and cancer cell death (Figure 5). ILs are the most important cytokines, aggravating tumor growth via multiple mechanisms.

Notably, IL-1α acts as an “alarmin”, mediating the initial stages of sterile inflammation besides recruiting neutrophils and monocytes in response to tissue injury [228]. In malignancies, IL-1α activates the TAMs which in turn, releases multiple growth factors and cytokines that aggravate the tumor cell proliferation, angiogenesis besides suppressing the adaptive immune response [229]. In a phase I study, a human MAb targeted towards IL-1, mammalian actin binding protein 1 (MABP1) was administered to 16, chemotherapy non-responsive NSCLC sufferers, in a dose-dependent manner. Analysis revealed a 7.6 months median OS with a 57 days, PFS. The treatment efficacy exhibited a correlation with anti-EGFR pretreated patients (9.4 months median OS), who responded better than others (4.8 months OS). The observations were supported by higher mPFS (97 days) in the anti-EGFR pretreated sufferers than others (PFS: 78 days). A small size was the lacuna in this study, making it inconclusive [230]. Another cytokine, IL-2 was first described as an immuno-stimulatory factor for the expansion of activated effector T cells, but it was later described as an immuno-suppressor based on doses [231]. A phase III randomized multicenter trial screened the impact of subcutaneous low-dose IL-2 with standard chemotherapy, on OS in NSCLC patients. Surprisingly, there were no discernible differences in outcomes on the inclusion of IL-2 to chemotherapy [232].

## Factors affecting the immunotherapies successful outcomes

The NSCLC manifestation is intriguingly affected by multiple oncogenes with the coordinated functioning of cytokines and chemokines, together exhibiting a key role in tumor growth and angiogenesis [233, 234]. In highly sensitive redox status, the actions of transcription factors and signaling proteins at the genetic and epigenetic platform critically regulate the tumor angiogenesis and metastasis. Prominent signaling pathways regulating cell proliferation and survival include MAPKs, AKT (a serine/threonine kinase) and nuclear factor kappa B (NF-κB) [235–237], which are impaired and ultimately aggravate the tumor growth. The transcription factor NF-κB is a key activator of tumor microenvironment, controlling the expression of several proto-oncogenes in murine models and humans [238–240]. NF-κB is rigorously implicated in setting up pro-tumor responses via recruiting the immunosuppressive cells, including the T_reg_ cells and myeloid DCs (mDCs). These cells release chemokines and cytokines alongwith the growth factors (e.g., VEGF) which are critically implicated in tumor growth and angiogenesis. Optimum NF-κB expression is essential as studies report its mutations driven aggravated angiogenesis and metastasis. Apart from NF-κB, type I interferons (IFNs, including α, β and γ) play critical roles in immunosurveillance and T-cell priming (that manifests a cytotoxic response). Studies report a key role of IFN-NF-κB crosstalk in setting up the tumor growth suppressing microenvironment [241, 242].

Antitumor functions of NF-κB are attributed to its signaling that recruits the T-cells in the tumor cell vicinity, leading to tumor regression apart from cytokine and chemokine activation harbouring the C-C and chemokine (C-C motif) ligand 2 (CCL2) motifs [243]. A recent study has reported an unusually aggravated CCL4 expression in NSCLC patients, with certain methylation features. The investigators noted CCL4 implication in the infiltration of immune cells (including B and CD8^+^ T cells). These findings gathered support from the single cell sequencing observations, exhibiting a high CCL4 expression in CD8^+^ T cells and regulated the generation pacing. Apart from this, CCL4 expression was screened as positively associated with PD-1 and PD-L1 functioning as well as mutated *EGFR*, *ALK* and *ROS1* associated with NSCLC treatment. So, the tumor suppressing and encouraging functions of NF-κB manifest a curiosity wherein the activities of Toll like receptors (TLRs), intercellular adhesion molecule 1 (ICAM-1) and lymphotoxin beta (LTB) assume significance [244].

Together, above mentioned mediators and immunologically significant genes play decisive roles in setting up NF-κB inflammatory and immunological responses, opening multiple research inclinations alongside a more reliable prognosis of LUAD. It is perhaps due to these rationalities only, that several targeted immunotherapeutic mechanisms exploit the tumor cell inhibiting responses of NF-κB. This is so as the tumor manifestation commences from the abnormal microenvironment (the home for intricate crosstalk amongst diverse signaling proteins, chemokines, cytokines and genes), instigated via NF-κB activation driven inflammatory stress, suggesting a suitability of anti-inflammatory drugs for cure [241, 245]. For instance, chemokine C-X-C chemokine receptor type 4 (CXCR4) plays a decisive role in NSCLC metastasis, forming an important constituent of tumor microenvironment [246]. The CXCR4 is implicated in the functions of pleural spaces with its expression extents being correlated with the C-X-C motif ligand 12 (CXCL12) chemokine expression, a key prospect of advanced stage NSCLC. Multiple evidences report an incessant CXCL12 chemokine expression on stromal, neoplastic, vascular and endothelial cells in stage I and II LUAD sufferers [233, 234, 247]. Correlation between CXCL12 expression and CXCR4 (inferred via CXCL12 expression) is known to aggravate the tumor growth via ERK pathway activation. Together these tumor growth promoting chemokines with supporting T_reg_ cells act in paracrine and autocrine manners to gather the growth promoting and aggravating inflammatory cytokines, which aggravate the angiogenesis and tumor growth. Thereby, these activities collectively provide multiple therapeutic targets which can increase the success of targeted immunotherapies. Though T-cell proliferation is controlled jointly by CTLA-4 and PD-1, more recent studies have reported the regulation of T-cell mediated immunity by the NF-κB activation, thereby deciphering their involvement in tumor cell immunosurveillance [248]. Some interesting findings in the recent studies have reported the mutations curtailing the efficacy of targeted immunotherapy and the molecular associations implicated in enhanced success thereof. Of note, the primary immunotherapeutic targets in NSCLC treatment include interleukins, CTLA-4 and PD-1/PD-L1. A recent study for instance, screened the reliable markers for predictive success of immunotherapy in NSCLC patients. Assessing the ICI over the cohort comprising of 240 patients, the investigators monitored copy number variations in the genes targeted in immunotherapy. Analyzing the immune biomarkers and immune infiltration in The Cancer Genome Atlas (TCGA)-NSCLC and the Gene Expression Omnibus (GEO) cohorts, the investigators noted significant PFS improvement subsequent to ubiquitin protein ligase E3A (UBE3A) erasure. Inspection revealed a higher immunocyte infiltration and expression of immune checkpoint biomarkers which modulated the enrichment extents of immune signaling pathways. Thus, the study demonstrated UBE3A removal as an explicit predictive NSCLC biomarker for the patients who are undergoing ICI therapy [249].

The working mechanism of immunotherapy relies on engineering the cytotoxic activity of immune cells, mediated via drifting across the troubled microenvironment of tumors. The resistant response towards chemotherapy is majorly attributed to the aggressive expression of receptor proteins in the tumor vicinity which obstructs the passage of CDs towards inside the tumor cells. With the continued findings of simultaneous chemo and immuno therapies, the success rate of tumor treatments have significantly improved but the real challenge remains to identify or detect them at the initial stages so that the treatment recourses could be optimized as preventive rather than the conventional curative regimes. Lately though, some interesting associations of some characteristic gene mutations have revealed enhanced success for immunotherapies. To follow up with these latest developments, the following paragraphs shed some light on these mutations and combinatorial attempts.

### Characteristic gene mutations as immunotherapy success predictors

Lately, the association of some characteristic mutated gene expressions have been reported distinctly for the success of immunotherapy. This section discusses a few recent attempts (mostly from 2022) reporting a comparatively higher efficacy with characteristic gene mutations prevailing natively or amidst chemotherapy. The first study of note is an attempt by Liu and colleagues [250] who noted KRAS-G12D mutation responsible for suppressing the PD-L1 targeted therapy and CXCL10/CXCL11 (chemokines) secretion, together suppressing the CD8^+^ tumor infiltrating lymphocytes (TILs). Analyzing the KRAS-G12D mutation exhibiting cell lines, the investigators noted a corresponding suppressed extent of PD-L1 via 70 kDa ribosomal protein S6 kinase (P70S6K)/PI3K/AKT axis and suppressed CXCL10/CXCL11 levels by inhibiting the high mobility group protein A2 (HMGA2) expression levels. The observation was counter-screened by co-delivering PD-L1 blockade with paclitaxel (alone being an HMGA2 upregulation and concomitant stimulation of CXCL10/CXCL11), revealing a decreased tumor growth compared to singular treatment with a PD-L1 inhibitor, in the KRAS-G12D-mutant LUAD carrying mouse model. Screening the distinction in the co-delivery mode, it was noticed that paclitaxel with PD-L1 inhibitor significantly engaged the CD8^+^ TILs towards enhanced CXCL10/CXCL11 expression extents. The study, therefore, clarified the mechanism by which KRAS mutant NSCLC exhibit resistance to immunotherapy, noticing a better response of paclitaxel + ICI-mediated treatment (than only ICIs) in KRAS-G12D mutant NSCLC sufferers [250].

A nearly similar study by Landre and associates [251] examined IMpower-150, KEYNOTE-189, and KEYNOTE-042 first-line and OAK, POPLAR, CHECKMATE 057, second-line clinical trials. These trails together comprised 386 KRAS mutant and 927 KRAS wild-type tumors. Analysis revealed anti-PD-L1 treatment with or without chemotherapy in KRAS mutant NSCLC sufferers to exhibit an enhanced OS (0.59) and PFS (0.58), compared to singular chemotherapy, in both clinical trials. A striking coincidence was the observation of enhanced OS and PFS only in KRAS mutant NSCLC but not in KRAS wild-type tumors. Thereby, the results explained a better response of singular and chemotherapy combined anti-PD-L1 therapies, for KRAS mutant and wild-type NSCLCs, with a higher OS for the mutant tumors [251]. Interesting mutational correlations were also demonstrated by Ricciuti and associates [252], who screened the cohort of 1,552, PD-1/PD-L1 blockade-received NSCLC patients, of which 830 were females and 1,347 had non-squamous histology. Analysis revealed a 57% ORR for PD-L1 inhibition in the sufferers having high tumor mutational burden (TMB) and 50% (or greater) PD-L1 expression and a mere 8.7% for those having low TMB and < 1% PD-L1 expression. The observations led the investigators to correlate the TMB expression extents with immune cell infiltration and inflammation-mediated T-cell mediated response, together enhancing the sensitivity to PD-1/PD-L1 blockade in the NSCLC cells expressing PD-L1 [252].

### Combinatorial therapies of ICIs

Combinatorial therapies have been the choice in the recent past to overcome the eventual resistant outcomes in monotherapeutic programmed schedules. For checkpoint inhibitors (CKIs), this realization gathers support from the likely additive or synergistic outcomes via enhancing the blockade of immune cells inhibition, using 2 distinct CKIs. The salient observations of such attempts employing PD-L1 and CTLA-4 pathway-targeting agents are summarized in the following paragraphs.

#### Durvalumab with tremelimumab

Tremelimumab is a CTLA-4 checkpoint inhibitor but currently, it is being remotely evaluated as a monotherapeutic anti-NSCLC agent. In a noted phase Ib trial, 102 immunotherapy naive, NSCLC sufferers were administered with a durvalumab + tremelimunab in a dose-increasing manner. Analysis revealed that 20 mg/kg durvalumab with 1 mg/kg tremelimumab every 4 weeks, is the optimum regimen for the expansion phase in terms of safety and clinical efficacy [253]. Another recent phase II trial monitored the response of 78 sufferers who were randomly administered durvalumab and tremelimumab, alone and in synergy with a low dose and hypofractionated radiotherapy. Inspection revealed grave AEs in merely 4% sufferers administered durvalumab + tremelimumab. Contrary to this, the proportion of AEs was 19% in the sufferers receiving additional low-dose radiotherapy and 15% in those having administered hypofractionated therapy. The findings eventually led to the conclusion that radiotherapy is unable to improve the outcomes of PD-L1 and CTLA-4 combinative therapies in NSCLC sufferers resistant to PD-1 and PD-L1 therapies [254]. An erstwhile phase III ARCTIC study examined the NSCLC patients having wild-type EGFR and ALK, having received a minimal two therapies (one being platinum chemotherapy). The sufferers were distinguished in group A having PD-L1 expression on a minimum of 25% tumor cells. This group was randomly conferred durvalumab or chemotherapy (as erlotinib, gemcitabine, or vinorelbine), wherein analysis revealed an OS and PFS of 11.7 months and 3.8 months for durvalumab while similar extents for chemotherapy were 6.8 months and 2.2 months. Group B of this study comprised the patients with wild-type PD-L1, who randomly received durvalumab + tremelimumab or chemotherapy (erlotinib, gemcitabine or vinorelbine). Analysis revealed the OS and PFS of 11.5 months and 3.5 months for durvalumab + tremelimumab, marginally better than 8.7 months and 2.2 months for chemotherapy. A significant observation of this attempt was the higher efficacy of durvalumab + tremelimumab, noticed as enhanced OS and PFS [254].

#### Nivolumab with ipilumab

This is a combinatorial regime comprising chemo (nivolumab) and immuno (ipilumab, an anti-CTLA-4 drug) therapeutic molecules. The combination was examined in open-label, phase III trials in 103 hospitals in 19 countries. Examined subjects included the treatment naïve individuals, histologically confirmed for recurrent NSCLC. The sufferers were randomly assigned vide an interactive web response provision via permuted blocks to nivolumab (360 mg intravenously every 3 weeks) alongwith ipilumab (1 mg/kg intravenously every 6 weeks) alongwith histology-driven platinum chemotherapy (intravenously every 3 weeks for 2 cycles, experimental group) and chemotherapy alone (every 3 weeks for 4 cycles, control group). Tumor histology, sex, and PD-L1 expression served as randomization criteria. The study is active (registered as NCT03215706) but no longer recruiting any patients. Analysis revealed a longer OS in all members of the experimental group than the control group (median 14.1 months against 10.7 months with 0.69 as the hazard ratio, all estimated at 95% CI). Frequent grade 3 to 4 TEAEs were neutropenia (in *n* = 24 experimental and *n* = 32 control groups), anaemia (*n* = 21 and *n* = 50), and diarrhoea (*n* = 14 and *n* = 10). Serious TEAEs were noticed in 30% sufferers in the experimental and 18% of the control group. Both groups responded with seven and six (accounting for 2%) treatment-related deaths. Findings on the whole demonstrated a significant OS improvement in nivolumab plus ipilimumab compared with two cycles of chemotherapy, supporting a first-line treatment regimen for NSCLC sufferers [255].

**Figure 6:**
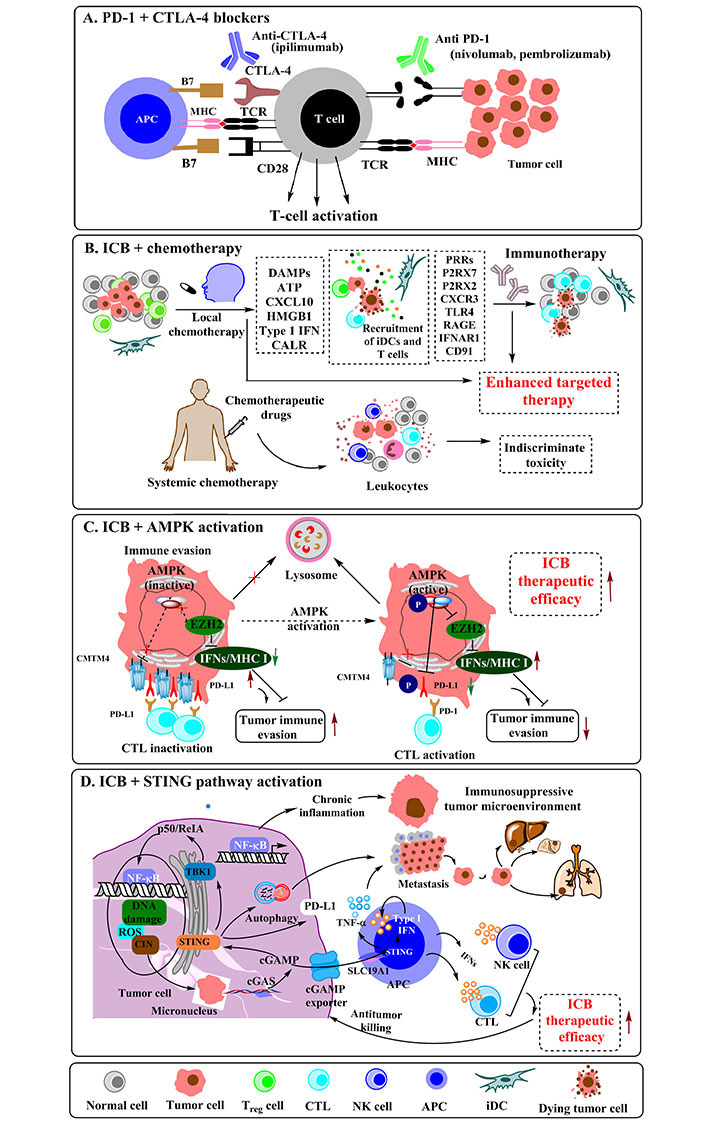
PD-1/PD-L1 based combinatorial therapies for enhanced NSCLC inhibiting efficacy. A. Combination therapy with PD-1 and CTLA-4 blockers. Simultaneous administration of PD-1 and CTLA-4 inhibitors mediates a synergistic response; B. combination therapy of immune-checkpoint blockers and chemotherapy. Chemotherapy induces ICD, stimulates the release of tumor antigens and DAMPs, activating DCs and stimulating the generation of CXCL10 and movement of T-cells to tumor bed for enhanced differentiation of antitumor-specific CTLs. While systemic therapy remains identical toxicity to tumor cells and anticancer immune system, the localized chemotherapy augments the immunotherapy via remodelling the tumor microenvironment besides relocating activated immune cells to the tumor region; C. AMPK activator comprising combination therapy for reduced PD-L1 levels in the presence of AMPK activation that enhances the treatment efficacy; D. STING agonists based combination therapy for NSCLC, cGAS-STING pathway links the innate and adaptive immunity against tumor cells. Cancer cells can bypass the immune surveillance via inactivating the cGAS-STING pathway, boosting the immunotherapy efficacy via combining ICB with STING agonists. DAMPs: damage-associated molecular patterns; CALR: calreticulin; iDCs: interdigitating DCs; P2RX7: purinoceptor, a therapeutic target; IFNAR1: IFN alpha and beta receptor subunit 1; CMTM4: a protein which promotes LC progression by stabilizing PD-L1; EZH2: enhancer of zeste homolog 2; P: phosphorylated state; p50/ReIA: dimer proteins stabilizing ubiquitination of IκB; CIN: cervical intraepithelial neoplasia; TBK1: TANK-binding kinase 1; cGAMP: cyclic guanosine monophosphate adenosine monophosphate; SLC19A1: solute carrier family 19 member 1; TNF-α: tumor necrosis factor-alpha

An ongoing study is screening 422 chemotherapy-naive NSCLC patients, randomly standardized in 1:1 proportion for receiving platinum chemotherapy and either pembrolizumab or nivolumab with ipilumab. The primary endpoint of this investigation is listed as OS although the results would be examined for their explicit association with therapeutic effects and AEs. Subject to validation of this trial, nivolumab + ipilimumab with platinum chemotherapy could be adopted as an efficacious standardized advanced NSCLC therapy [256]. Thereby, the combined delivery of two immune CKIs has enabled a better response to date, anticipating optimism for future trials. Realizing the dearth of reliable metastatic squamous or non-squamous NSCLC therapies, Zhou and colleagues [257] examined the efficacy of sugemalimab (a PD-L1 inhibitor) combined with chemotherapy on metastatic squamous or non-squamous NSCLC patients via randomized, phase III clinical trial, GEMSTONE-302. Of note, the combinatorial efficacy of PD-1 inhibitor and chemotherapy as first-line treatment for metastatic NSCLC patients is well-demonstrated. Analysis revealed statistically significant benefits in terms of PFS for sugemalimab with chemotherapy compared to placebo-accompanied chemotherapy in the untreated squamous and non-squamous NSCLC sufferers, irrespective of PD-L1 expression. The results offered significant betterment towards NSCLC management of squamous and non-squamous metastatic NSCLC, in first-line settings [257]. The PD-1/PD-L1 based combinatorial NSCLC therapies primarily relies on simultaneous PD-1 and CTLA-4 inhibition (Figure 6). The mechanism involves the actions of activated T-cells which express CTLA-4, PD-1, T-cell receptors and CD28. Next combinatorial strategy uses CDs in combination with ICBs. The receptors implicated in local chemotherapy and immunotherapy, modulate the synergistic responses. From the listed options, CXCL10 and CXCR3 are the characteristics of NSCLC while receptor for advanced glycation end products (RAGE) and its ligand, high mobility group box-1 protein (HMGB1) could contribute discretely in activating the immuno and chemotherapy. Another option for combinatorial approach involves AMP-activated protein kinase (AMPK) activator based combination therapy which aggravates the PD-L1 via simultaneous AMPK activation. The last approach uses stimulator of *IFN* gene (STING) agonists, using the cyclic GMP-AMP synthase (cGAS)-STING pathway for stimulating the adoptive and innate immunity against the cancers. Combining ICB with STING agonists forbids the escape of cancer cells by the immuno-surveialance to enhance the immunotherapy efficacy.

## Conclusions

Despite significant awareness, resistant responses to chemotherapies continue to curtail the NSCLC treatment, a claim stoutly supported by the fact that most of the CDs for vulnerable mutations have reached the development phase of 4th generation. Resistant outcomes are majorly due to singular targeted programming of CDs whereby if the targeted site is attenuated due to genetic changes, there is no bypass alternative. This limitation is being intensively resolved by the two prominent strategies, with the more common route focused on nanocarrier-mediated delivery. Irrespective of the composition and constitution, all nanocarriers are designed to confer structural protection to the trafficked drugs so that their therapeutic potency is prolonged and the site-specific action is facilitated. The moderate binding forces catalyze a sustainable transport of the drugs by their large surface areas and immense functionalization abilities. Of the various nanocarriers, Au and Ag nanoparticles are being screened with specific interest due to their robust preparation methods. The second reason is the fact that using nanoparticles, both active and passive delivery mechanisms could be optimized [258, 259]. Besides nanocarrier-mediated delivery, a mechanism gathering interest of late involves the combinatorial delivery of CDs with pleiotropic natural compounds. Optimizing these combinations with a higher proportion of polyphenols guards against instantaneous toxic response and enhances the tumor cell internalization of CDs [260]. Several polyphenols have been screened for their anticancer activities which offer interest due to simultaneous actions at multiple proteins and signaling pathways. The combinatorial delivery could be primed for two or more CDs but often distinct drug categories are chosen so that the toxicity is moderated and self-interactions are minimized. For NSCLC in particular, the recent success of mutated gene-programmed CDs being co-delivered with immunotherapeutic drugs is quite encouraging [251, 252, 255–257]. Co-delivery via nanocarriers exhibits distinction by conferring enhanced structural protection to the CDs, a prospect strongly supported by lower IC_50_ than solitary mode.

Understanding gene mutations in cancer is a comprehensive task whereby expertise from multiple disciplines is needed. A primitive requirement in this context involves the timely detection of breeding tumors whereby prompt treatments and optimum drug delivery regimes could be exercised. This conditioning mandates accurate sensing of the tumor, for which nanomaterials are being used to improve the detection and distinction sensitivity of biosensors. With their significant shape and size modulated physicochemical properties, the nanomaterials like carbon nanotubes, immobilized nanoparticles and graphene functionalized nanoassemblies are being attempted to detect the pico and nano molar extents of analytes alongside their infinitesimal interaction sensitivity [261]. Secondly, it would be a boost if combinatorial attempts involving ICIs swiftly progress to clinical trials and further validation as immunotherapy amalgamation has been a boost to CDs efficacy. An urgent need of maintaining a robust database is the need of hour. A rigorous database collection and a steadfast tracking of clinical trials with smoking habits and the reproducibility prospects could certainly boost the success of NSCLC treatments.

## Abbreviations

ADC: antibody-drug conjugate
AEs: adverse events
AKT: protein kinase B
ALK: anaplastic lymphoma kinase
AMPK: AMP-activated protein kinase
APCs: antigen-presenting cells
BRAF: v-RAF murine sarcoma viral oncogene homolog B1
CCL2: chemokine (C-C motif) ligand 2
CDs: chemotherapeutic drugs
CEP7: chromosome 7 centromere
cGAS: cyclic GMP-AMP synthase
CI: confidence interval
CNS: central nervous system
CPR: complete pathologic response
CSAs: cancer-specific antigens
CTLA-4: cytotoxic T-lymphocyte associated antigen-4
CTLs: cytotoxic T lymphocytes
CXCL12: C-X-C motif ligand 12
CXCR4: C-X-C chemokine receptor type 4
DCR: disease control rate
DCs: dendritic cells
DOR: duration of response
EGFRs: epidermal growth factor receptors
EM: skipping mutation
EML4: echinoderm microtubule-associated protein-like 4
ERKs: extracellular signal-regulated kinases
FDA: Food and Drug Administration
GCN: gene copy number
GTP: guanosine triphosphate
HER1: human epidermal growth factor receptor 1
IC_50_: half maximal inhibitory concentration
ICB: immune-checkpoint blockade
ICIs: immune checkpoint inhibitors
IFNs: interferons
IgG1: immunoglobulin G1
KIF5B: kinesin family member 5B
KRAS: Kirsten rat sarcoma
LC: lung cancer
LUAD: lung adenocarcinoma
mAbs: monoclonal antibodies
MAPK: mitogen-activated protein kinase
MEK5: mitogen-activated protein kinase 2/3 mitogen-activated protein kinase 5
MET: mesenchymal-epithelial transition
mPFS: median progression-free survival
MPR: major pathologic response
mTOR: mammalian target of rapamycin
NF-κB: nuclear factor kappa B
NSCLCs: non-small cell lung cancers
NTRK: neurotrophic tropomyosin receptor kinase
OD: once a day
ORR: overall response rate
OS: overall survival
PD-1: programmed cell death-1
PD-L1: programmed cell death-1 ligand
PFS: progression-free survival
PI3K: phosphatidylinositol 3-kinase
PR: partial response
PSC: pulmonary sarcomatoid carcinoma
RAS: rat sarcoma
RET: rearrangements during transfection
ROS1: Proto-oncogene tyrosine-protein kinase
RTKs: receptor tyrosine kinases
SHP2: Src homology phosphotyrosine phosphatase 2
STAT3: signal transducer and activator of transcription 3
STING: stimulator of interferon gene
TAMs: tumor-associated macrophages
T_c_: cytotoxic T cells
TEAEs: treatment emergent adverse events
TKI: tyrosine kinase inhibitor
TMB: tumor mutational burden
T_reg_: T regulatory
TRK: tropomyosin receptor kinase
VEGF: vascular endothelial growth factor
xDFG: mutations stabilizing inactive kinase conformation

## Declarations

### Copyright

© The Author(s) 2023.
